# The Roles of GSK-3β in Regulation of Retinoid Signaling and Sorafenib Treatment Response in Hepatocellular Carcinoma

**DOI:** 10.7150/thno.38711

**Published:** 2020-01-01

**Authors:** Shuaishuai Zhang, Weiwei Gao, Juan Tang, Huaifang Zhang, Yuqi Zhou, Jie Liu, Kun Chen, Fangzhou Liu, Wengang Li, Sally K. Y. To, Alice Sze Tsai Wong, Xiao-kun Zhang, Hu Zhou, Jin-Zhang Zeng

**Affiliations:** 1 State Key Laboratory of Cellular Stress Biology and Fujian Provincial Key Laboratory of Innovative Drug Target Research, School of Pharmaceutical Sciences, Xiamen University, Xiang'an, Xiamen 361102, China; 2 The 174 th Hospital, Xiamen University, Xiang'an, Xiamen 361102, China; 3 School of Biological Sciences, University of Hong Kong, Pokfulam Road, Hong Kong

**Keywords:** Retinoid Receptor, GSK-3β, Hepatocellular Carcinoma, Target Therapy

## Abstract

**Rationale**: Glycogen synthase kinase-3β (GSK-3β) plays key roles in metabolism and many cellular processes. It was recently demonstrated that overexpression of GSK-3β can confer tumor growth. However, the expression and function of GSK-3β in hepatocellular carcinoma (HCC) remain largely unexplored. This study is aimed at investigating the role and therapeutic target value of GSK-3β in HCC.

**Methods**: We firstly clarified the expression of GSK-3β in human HCC samples. Given that deviated retinoid signalling is critical for HCC development, we studied whether GSK-3β could be involved in the regulation. Since sorafenib is currently used to treat HCC, the involvement of GSK-3β in sorafenib treatment response was determined. Co-immunoprecipitation, GST pull down,* in vitro* kinase assay, luciferase reporter and chromatin immunoprecipitation were used to explore the molecular mechanism. The biological readouts were examined with MTT, flow cytometry and animal experiments.

**Results**: We demonstrated that GSK-3β is highly expressed in HCC and associated with shorter overall survival (OS). Overexpression of GSK-3β confers HCC cell colony formation and xenograft tumor growth. Tumor-associated GSK-3β is correlated with reduced expression of retinoic acid receptor-β (RARβ), which is caused by GSK-3β-mediated phosphorylation and heterodimerization abrogation of retinoid X receptor (RXRα) with RARα on RARβ promoter. Overexpression of functional GSK-3β impairs retinoid response and represses sorafenib anti-HCC effect. Inactivation of GSK-3β by tideglusib can potentiate 9-*cis*-RA enhancement of sorafenib sensitivity (tumor inhibition from 48.3% to 93.4%). Efficient induction of RARβ by tideglusib/9-*cis-*RA is required for enhanced therapeutic outcome of sorafenib, which effect is greatly inhibited by knocking down RARβ.

**Conclusions**: Our findings demonstrate that GSK-3β is a disruptor of retinoid signalling and a new resistant factor of sorafenib in HCC. Targeting GSK-3β may be a promising strategy for HCC treatment in clinic.

## Introduction

Glycogen Synthase Kinase-3 (GSK-3) is a proline-directed serine/threonine kinase with two ubiquitously expressed homologous isoforms, GSK-3α and GSK-3β, in mammal cells 1, 2. GSK-3β is constitutively active in resting cells essential for cellular metabolism and homeostasis in normal tissues. GSK-3β is known to suppress tumor growth as it negatively regulate Wnt/β**-**catenin, PI3K/AKT and many other oncogenic pathways 3, 4. Inversely, GSK-3β can be phosphorylated and inactivated by AKT and sequestrated in multi-vesicular endosomes by Wnt signalling. However, many tumors highly express functional GSK-3β in irrespective of AKT and Wnt activation 5, 6. Overexpression of GSK-3β can facilitate tumor growth and there are considerable evidence to show that GSK-3β is rationally a therapeutic target 7, 8. Down-regulation of GSK-3β by siRNA and microRNA-129 or pharmacological inhibition of GSK-3β with lithium chloride, indirubins, 9-ING-41 and 9-ING-87 can inhibit tumor cell growth and metastasis 9-12.

Retinoids are very important for hepatic homeostasis, which effects are mediated by retinoic acid receptors (RARα, RARβ and RARγ) and retinoid X receptors (RXRα, RXRβ and RXRγ) 13. Deregulated metabolism of retinoids and altered expression of their receptors are implicated in HCC development and progression 14, 15. GSK-3β can inhibit RARα-dependent differentiation of myeloid leukemia 16, 17. Paradoxically, GSK-3β protects RXRα from calpain-mediated truncation in certain solid tumors 18. Implication of GSK-3β in retinoid signaling and HCC development need further explore. We characterized here RXRα as a direct substrate for GSK-3β. GSK-3β phosphorylates RXRα and impairs its activation of RARβ promoter. Clinically, GSK-3β is overexpressed and associated with RARβ reduction in a majority of HCC. RARβ mediates retinoid action but is frequently silenced during carcinogenesis 14. Thus, GSK-3β may confer HCC through interfering RARβ-mediated retinoid signalling. This prompted us to further determine whether targeting GSK-3β/RARβ could be of therapeutic significance in HCC.

Sorafenib, a multi-kinase inhibitor, is currently used to treat HCC 19, 20. However, its therapeutic resistance remains a significant problem in clinic 21, 22. Interestingly, sorafenib can stimulate GSK-3β activity* in vitro* and *in vivo*. We demonstrated that GSK-3β regulation of RARβ is involved in sorafenib resistance in HCC.

## Materials and Methods

### Antibodies and Reagents

Antibodies include: anti-GSK-3β (D5C5Z, #12456), anti-phospho-GSK-3β (Ser9,5B3) (#9323) anti-Ki-67 (8D5, #9449) anti-Cleaved Caspase-3 (Asp175) (#9661) from Cell Signaling Technology (CST); anti-phospho-serine/threonine (ab15556) and anti-phospho-serine (ab9332) from Abcam; anti-Flag^®^ M2 from Sigma-Aldrich; anti-RARB (A1603) from ABclonal; anti-PARP1 (66520-1-Ig) and anti-His (66005-1-Ig) from Proteintech; anti-rabbit and anti-mouse secondary antibodies conjugated to horseradish peroxidase, anti-rabbit secondary antibodies conjugated to Cy3 from Invitrogen; anti-RXRα (ΔN197, sc-774; D20, sc-553), anti-RARα (C20, sc-551), anti-GFP (B-2,sc-9996), anti-c-Myc (9E10, sc-40), anti-β-actin (H196, sc-7210) and anti-GAPDH (FL-335, sc-25778) from Santa Cruz Biotechnology. Chemicals and other regents are: lipofectamin 2000 from Invitrogen; enhanced chemiluminescence (ECL), protein A/G agarose from ThermoFisher Scientific; sorafenib, BIO, SB415286 and tideglusib from Selleck Chemicals; 3-(4,5-Dimethyl-2-thiazolyl)-2,5-diphenyl-2H-tetrazolium bromide (MTT), 4,6-Diamidino-2-phenylindole (DAPI), 9-*cis*-RA, isopropyl-1-thio-b-D-galactopyranoside (IPTG) and LiCl from Sigma-Aldrich; cocktail of proteinase and phosphatase inhibitors from Roche; Dual-Luciferase Assay System Kit from Promega.

### HCC samples

HCC samples (tumors and para-tumor tissues) were collected from The 174^th^ Hospital affiliated to Xiamen University. The tumors were histologically diagnosed as described 23, 24. All the use of human samples and study protocols were approved by the Hospital Ethics Committee. All patients signed an informed consent form in prior to sample collection. The clinical data were provided in Table S1.

### Cell culture and transfection

HepG2 (HB-8065) and HEK293T (CRL-11268) were purchased from ATCC, while SMMC-7721, Bel-7402, and QGY-7703 from Institute of Biochemistry and Cell Biology (SIBS, CAS). All cell lines were obtained between 2008 and 2013 and authenticated by the vendors. The newly received cells were expanded and aliquots of less than 10 passages were stored in liquid nitrogen. All cell lines were kept at low passage, returning to original frozen stocks every 6 months. During the course of this study, cells were thawed and passaged within 2 months in each experiment. QGY-7703 was cultured in RPMI-1640 medium, while other cell lines were grown in Dulbecco's Modified Eagle's Medium. The cultured cells were supplemented with 10% fetal bovine serum. Sub-confluent cells with exponential growth were used throughout the experiments. Transfections were carried out by using Lipofectamine 2000 according to manufacturer's instructions.

### Generation of stable lines

GSK-3β stable lines were generated with retroviral vectors. Briefly, HEK293T cells were transfected with PCDH-puro-GSK-3β together with envelope plasmid VsVg (addgene, #8454) and packaging plasmid psPAX2 (addgene, #12260). Retroviral supernatant was harvested at 48 h after initial plasmid transfection and then infected various HCC cell lines. Stable cell pools were selected with 1 μg/ml puromycin (Amresco). The expression efficiency was determined by Western blotting and RT-PCR.

### MTT assay and flow cytometry

Cell viability was performed with MTT method as described 15. For apoptotic analysis, control and treated cells were harvested and washed with precooled PBS twice. The cells were then stained with Annexin V-FITC and propidium iodide (PI) at room temperature for 15 min in the dark. Apoptotic cells were quantitated with flow cytometer analysis (Thermo, Attune NxT).

### siRNA synthesis

The siRNA sequences for RARβ (AUA UUC UUC UGA AUA CUU CUG), for GSK-3β (GUA UUG CAG GAC AAG AGA UdTdT), and non-targeting sequence (UUC UCC GAA CGU GUC ACG UTT) were synthesized by Sangon (Shanghai).

### RXRα mutant constructs and βRARE reporter

pCMV-myc vector was used to clone a set of plasmids: N-terminal truncation of RXRα (∆N20, ∆N60, ∆N80, ∆N100) and its point mutations (S49A, S66A, T53A, S78A, T82A, and T82D). Luciferase reporter plasmid of pGL6-βRARE was constructed with RARβ gene's retinoic acid response element sequence (5'-GGG TAG GGT TCA CCG AAA GTT CAC TCG-3').

### Dual-luciferase reporter assays

Cells were co-transfected with pGL6-βRARE firefly luciferase reporter constructs, renilla luciferase expression vector (renilla), and ΗΑ-RARα/myc-RXRα in the presence or absence of GSK-3β. The cells were treated with 1 μM 9-*cis*-RA combined with or without 5 μM tideglusib for 20 h. Cell lysates were measured for luciferase activities. The fluorescence intensity was detected in Multiskan Spectrum (PerkinElmer, USA). The renilla luciferase activity was used to normalize for transfection efficiency.

### Co-immunoprecipitation

HCC cells and tumor tissues were lysed and sonicated in 500 μL lysis buffer containing 150 mM NaCl, 100 mM NaF, 50 mM Tris-HCl (pH 7.6), 0.5% NP-40 and 1 mM PMSF. The lysates were incubated with antibodies against endogenous or tags of ectopic proteins and purified with protein A/G beads. For detection of GSK-3β-associated RXRα phosphorylation, HCC tumor lysates were subjected to two rounds of immunoprecipitation (IP). The first round of IP (IP1) was performed with anti-RXRα (D20), which product was then subjected to secondary IP (IP2) with anti-GSK-3β antibody. The lysates or IP2 samples were separated by 10% SDS-PAGE and blotted with anti-p-S/T antibody.

### GST pull down

The constructs of pGEX-4T-1-GST-RXRα and pET-22b-His-GSK-3β were transformed into *E. coli*. BL21 and *E. coli*. BL21 (DE3) respectively. Both cells were induced with 1 mM IPTG at 25ºC for 16 h. Bacterial pellets were sonicated and centrifuged. The supernatants were mixed with Glutathione-Sepharose (Thermo Scientific) and Ni-NTA agarose (Qiagen), for purification of GST-RXRα and His-GSK-3β, respectively. Immobilized GST-RXRα protein was incubated with 1 μg His-GSK-3β protein in 500 μl of 150 mM NaCl, 100 mM NaF, 50 mM Tris-HCl, pH 7.6, 0.5% NP-40 and 1 mM PMSF at 4 °C for 2 h. After thoroughly washing, bound proteins were eluted with elution buffer (20 mM Tris-HCl, pH 8.0; 10 mM GSH; 1 mM PMSF; 5 mM DTT) and processed for SDS-PAGE.

### *In vitro* kinase assays

GFP-RXRα was expressed in and purified from HepG2 cells with immunoprecipitation (IP) using anti-GFP antibody. The cell lysates and IP products were incubated with bacterially purified His-GSK-3β protein in a kinase reaction buffer (pH 7.5, 20 mM Tris-HCl, 10 mM MgCl_2_ and 100 mM ATP) at 37ºC for 45 min. The reactions were stopped by boiling the samples in loading buffer for 10 min and then separated with 10% SDS-PAGE. GSK-3β-induced RXRα phosphorylation was detected by anti-phospho-ser/thr (p-S/T) antibody.

### Chromatin immunoprecipitation assay

Cells were cross-linked with 0.75% formaldehyde in PBS for 10 min and sonicated in lysis buffer (50 mM HEPES-KOH, pH 8.0, 140 mM NaCl, and 1% TritonX-100). Immunoprecipitation of the chromatin was performed with anti-RXRα (D20) or normal rabbit IgG in 1× dilution buffer (1.0% Triton-X-100, 2 mM EDTA, 150 mM NaCl, and 20 mM Tris-HCl, pH 8.0). Immunocomplexes were purified with A/G agarose beads (Thermo Scientific) and incubated with RNase A (0.5 mg/ml) at 65°C overnight to remove RNA contamination and reverse formaldehyde-induced cross-linking. DNA fragments were purified with a DNA purification kit (Axygen Incorporation, China) and subjected to real-time PCR analysis. The primers for PCR amplification were: 5'-GCT CTG TGA GAA TCC TGG GA-3' (Forward) and 5'-TGC CTC TGA ACA GCT CAC TT-3' (Reverse) located between -124~+37 bp on RARβ promoter.

### RT-PCR

Total RNA was extracted by Trizol (Transgen Biotech). Complementary DNA was synthesized using FastQuant RT Kit (TianGen, Beijing, China). PCR experiments were performed with 2×Hieff™ PCR Master Mix (Yeasen, Shanghai, China) following manufacturer's protocol. The primers for RARβ mRNA transcription were: 5'-TCT CAG TGC CAT CTG CTT AAT CTG-3' (Forward) and 5'-CCA GCA ATG GTT CTT GTA GCT TAT C-3' (Reverse); for GAPDH mRNA transcription: 5'-AGG TCG GAG TCA ACG GAT TT-3' (Forward) and 5'-TGA CGG TGC CAT GGA ATT TG-3' (Reverse).

### Animal experiments

Male BALB/c nude mice were injected with HepG2/3β cells (2×10^6^ cells) subcutaneously in the posterior flanks and treated with 10 mg/kg sorafenib, 2 mg/kg 9-*cis*-RA, and 5 mg/kg tideglusib every other day at Day 3 of post-implantation. After three weeks of treatment, the mice were sacrificed. The tumors and various organ tissues were collected for further analysis. Tumor volume was measured twice weekly with a caliper. Tumor samples were immunoblotted with antibodies against RARβ, GSK-3β, p-GSK-3β and GAPDH. Paraffin sections were immunostained using antibodies against Ki-67 and cleaved caspase 3 with DAB Detection Kit (Polymer) (MXB biotechnologies, Fuzhou, China). The expression of p-GSK-3β and RARβ was determined with fluorescent immunostaining. The sections were co-stained with DAPI and detected by Laser Scanning Confocal Microscope (Zeiss). The study protocols were approved by the Institutional Animal Care and Use Committee of University of Xiamen University.

### Statistical analysis

Data were represented as mean ± standard deviation (SD) or median ± SEM. The statistical significances of differences were determined using an analysis of variance or Student t test. A P value of <0.05 was considered as significant. All data were acquired in at least three independent experiments.

## Results

### GSK-3β is overexpressed and associated with RARβ reduction in HCC

**Figure 1:**
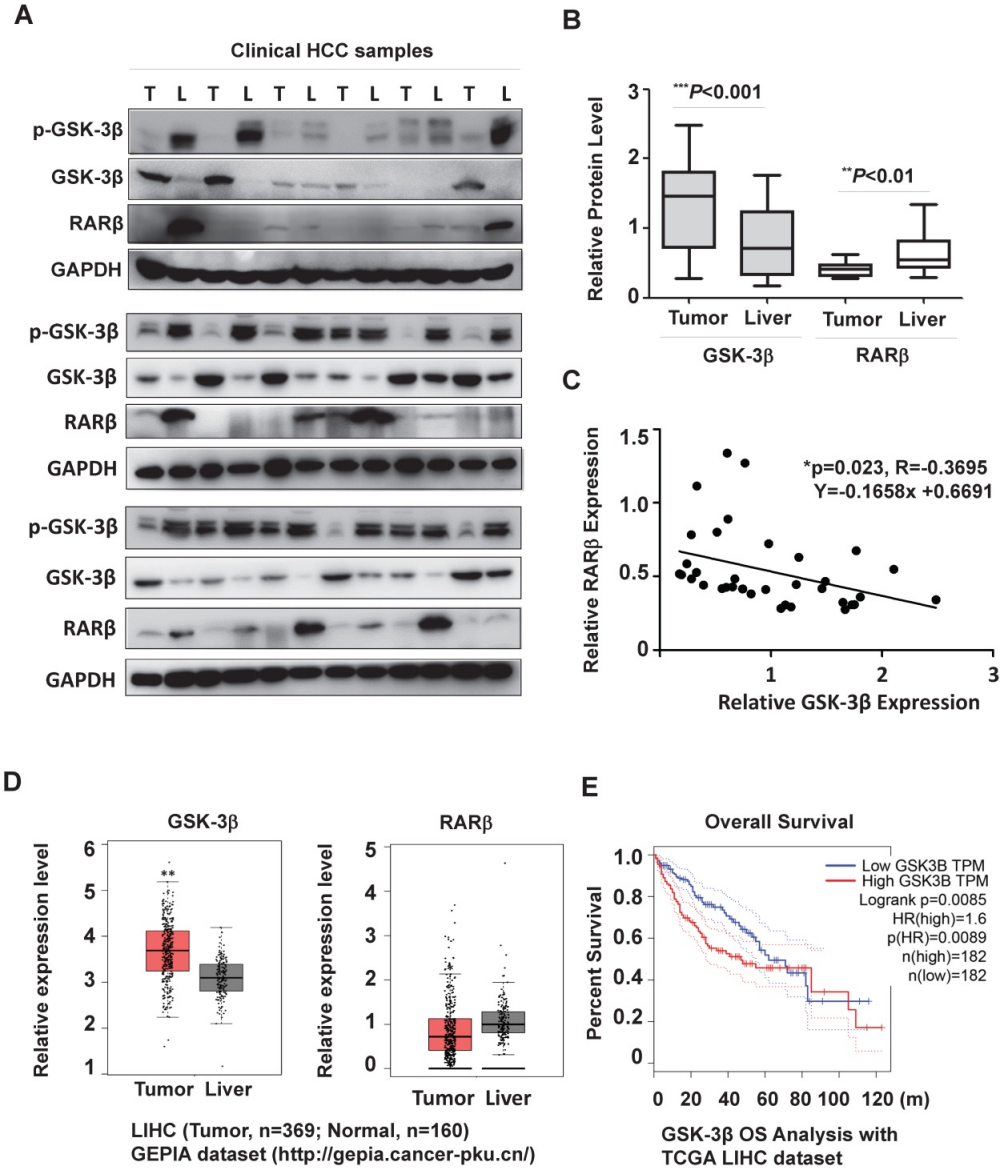
** GSK-3β is overexpressed and associated with reduced RARβ expression in HCC. (A)** The surgical samples were collected from 18 patients with HCC. GSK-3β, p-GSK-3β/Ser^9^ and RARβ expression were detected by western blotting in tumor (T) and adjacent liver (L) tissues. GAPDH was used as loading control.** (B)** The protein levels were calculated and normalized to GAPDH based on grey values using Quantity One software (Bio-Rad). The median levels of GSK-3β and RARβ were compared between tumor and nontumor tissues. *p* values are shown. **(C)** The correlation of GSK-3β with RARβ was determined by Pearson Correlation Analysis. **(D)** The clinical relevance of GSK-3β and RARβ in HCC were further analysed with GEPIA by using TCGA-LIHC database (http://gepia.cancer-pku.cn/). The median levels of GSK-3β and RARβ were compared between tumors (n=369) and liver tissues (n=160);^*^p<0.05 and^**^p<0.01 (Tumor *vs* Liver). **(E)** Overall survival (OS) rate analysis. The cut-off level of GSK-3β was set at median level. Based on it, GSK-3β expression levels were divided into high and low expression groups. The OS was compared between these groups. HR (Hazards Ratio) =1.6, p(HR)=0.0089.

To clarify the role of GSK-3β in HCC, we firstly collected HCC samples (n=18) to examine GSK-3β expression. Our results showed that GSK-3β was upregulated in 66.7% of tumors (≥1.5-fold increase) compared to adjacent liver tissues (Fig. 1A, 1B and Table S1). This was consistent with other reports 25, 26. In most tumors, increased GSK-3β expression remained significant active (with low Ser^9^ phosphorylation level) (Fig. 1A). We noted that high GSK-3β was not correlated to downregulation of total and nuclear β-catenin (Fig. S1A and B), suggesting that the tumor-suppressing effect of GSK-3β *via* Wnt/β-catenin was lost in HCC. To study whether GSK-3β could confer HCC growth, we overexpressed or knocked down GSK-3β with various HCC cell lines. We showed that overexpression of GSK-3β could strongly promote colony-forming capability of HCC cells, while siRNA-mediated downregulation of GSK-3β resulted in reduced colony formation (Fig. S1C). The role of GSK-3β in HCC was further strengthened in two HepG2/siβ clones (Fig. S1D). Importantly, GSK-3β-mediated tumor growth and proliferation was confirmed in *in vivo* experiment (Fig. S1E-G).

Interestingly, tumor-associated GSK-3β was inversely correlated with RARβ expression (Fig. 1A and C). To further evaluate the possible clinical relevance of GSK-3β/RARβ, we analyzed GEPIA (Gene Expression Profiling Interactive Analysis) database (http://gepia.cancer-pku.cn/). Agreement with our results, GSK-3β was higher and RARβ lower in HCC than adjacent liver tissue (Fig. 1D), which phenomenon was also observed in many other malignant tumors (Supplementary Fig. S2). Kalpen-Meier survival plot showed that the patients with high GSK-3β had a shorter overall survival (OS) than those with low GSK-3β (Fig. 1E). Together, our results suggest that GSK-3β play a role in RARβ regulation and HCC development.

### Identification of RXRα as GSK-3β substrate

**Figure 2:**
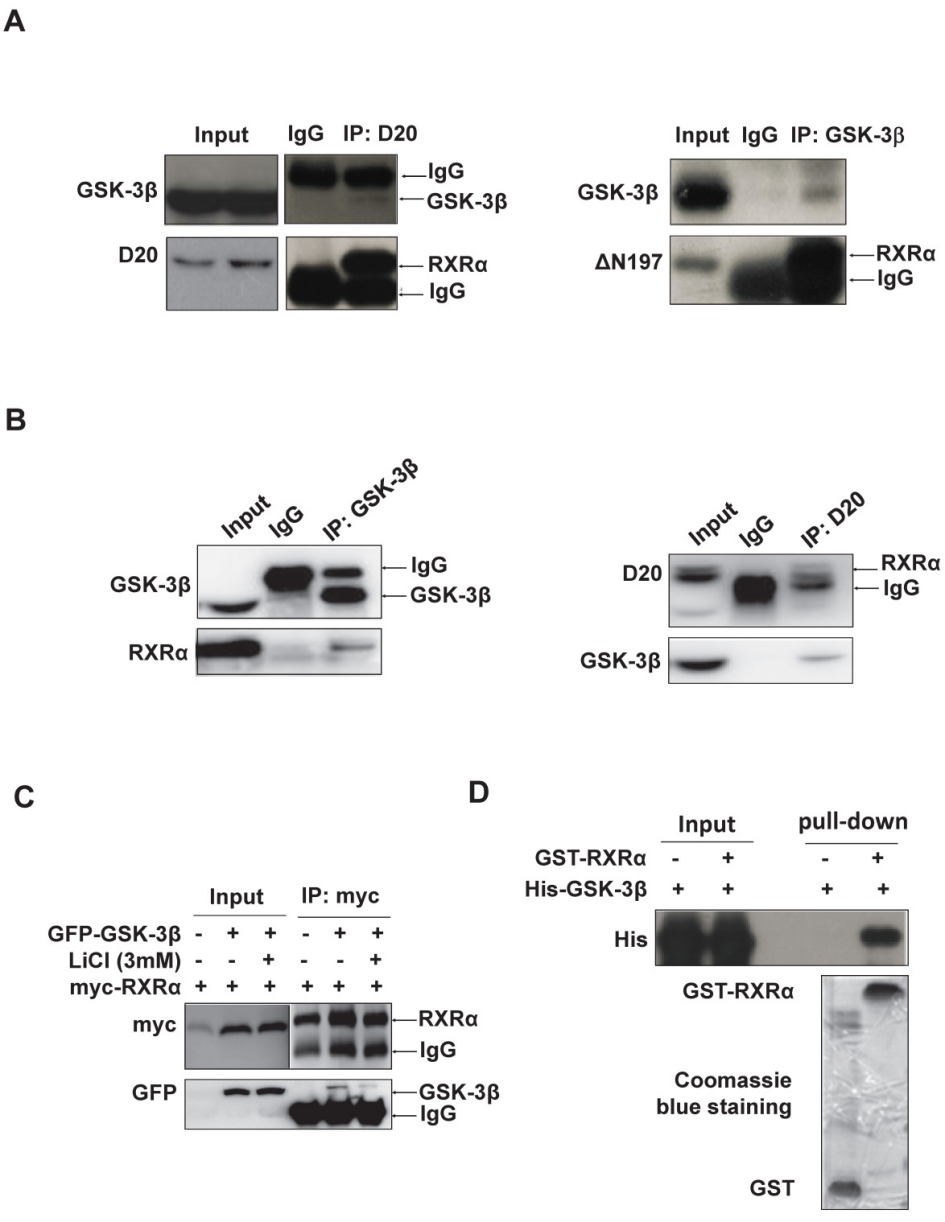
** GSK-3β interacts with RXRα. (A)(B)** The interaction between GSK-3β and RXRα was determined *via* co-IP. The tumor tissues (A) and HepG2 cells (B) were lysed and incubated with anti-RXRα (D20 or △N197) or anti-GSK-3β antibodies or nonspecific IgG. The IP samples was immunoblotted by anti-RXRα and anti-GSK-3β antibodies.** (C)** HEK-293T cells were transfected with myc-RXRα in the presence or absence of GFP-GSK-3β. The cells were treated with vehicle or 3 mM LiCl for 3 h. The cell lysates were incubated with anti-myc tag or with nonspecific IgG. For A, B and C, the inputs were loaded with 5% of each whole-cell lysate. **(D)** GST-pull down. GST and GST-tagged RXRα were purified with glutathione agarose beads and incubated with His-GSK-3β. The membranes were detected with anti-His antibody. GST and GST-RXRα expressions were indicated in coomassie blue staining.

RARβ expression was subjected to transcriptional regulation by RXRα 27. We thus investigated whether GSK-3β could directly interact with and regulate RXRα function. Indeed, our results showed that GSK-3β and RXRα were co-immunoprecipitated mutually in HCC tissues (Fig. 2A) and HepG2 hepatoma cells (Fig. 2B). When co-transfection, GSK-3β could interact with RXRα in HEK293T cells (Fig. 2C). Interestingly, the interaction between GSK-3β and RXRα could be abrogated when GSK-3β was inactivated by LiCl (Fig. 2C). Further, cell-free experiments showed that GST-RXRα could pull down His-GSK-3β demonstrating their direct association (Fig. 2D).

**Figure 3:**
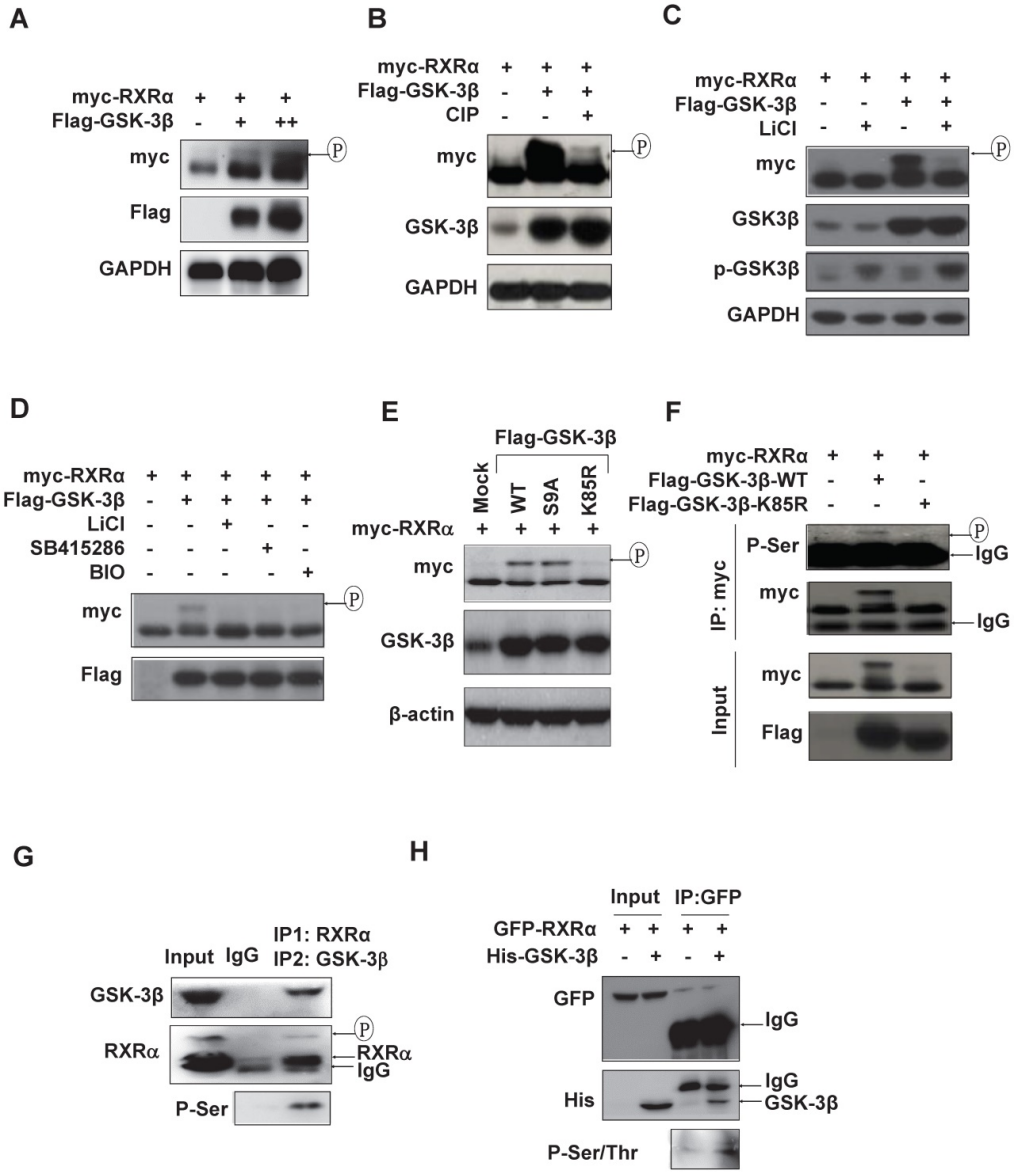
** RXRα is substrate of GSK-3β. (A)** HepG2 cells were co-transfected with myc-RXRα with or without increasing concentrations of Flag-GSK-3β. **(B)** HEK293T cells were co-transfected with Flag-GSK-3β and myc-RXRα. The lysates were incubated CIAP (0.2 U/mL) at 37ºC for 30 min. For A and B, the cells were lysed and subjected to IB analysis with anti-myc, GSK-3β, and GAPDH antibodies. Upshifted bands of RXRα indicated phosphorylation, which was confirmed below by anti-p-S/T antibody. **(C)(D)** HepG2 cells were transfected with myc-RXRα in the presence or absence Flag-GSK-3β. The cells were treated with vehicle or with different GSK-3β inhibitors of 20 mM LiCl, 10 μM SB415286, or 5μM BIO for 3 h. RXRα expression and phosphorylation statuses were detected with anti-myc, while GSK-3β expression and its Ser^9^ phosphorylation were documented with anti-Flag-GSK-3β and anti-phospho-GSK-3β respectively. **(E)** HepG2 cells were transfected with myc-RXRα in combination with or without Flag-tagged GSK-3β/wt, GSK-3β/S9A, or GSK-3β/K85R. The cells were lysed and blotted with anti-myc, anti-GSK-3β and anti-β-actin antibodies. **(F)** HEK293T cells were transfected with myc-RXRα alone or in combination with Flag-GSK-3β/wt or Flag-GSK-3β/K85R. The cells were lysed and immunoprecipitated with anti-myc tag antibody or IgG as a control. The IP products were detected for RXRα phosphorylation with anti-phospho-serine antibody. The input was 5% of whole cell lysates. **(G)** HCC tumor tissues were lysed and subjected to two rounds of immunoprecipitation. The lysates were immunoprecipitated with anti-RXRα antibody (D20) (IP1) and then with anti-GSK-3β antibody (IP2). The double IP products were blotted with anti-phospho-ser, anti-RXRα (D20) and anti-GSK-3β antibodies. **(H)** *In vitro* analysis. GFP-RXRα was purified from transfected HepG2 cells with GFP antibody, while His-GSK-3β was expressed and purified from *E. coli* BL21 (△E3) with His beads. GFP-RXRα and His-GSK-3β were incubated with 100 μM ATP for 3 h and blotted with anti-p-S/T, anti-GFP and anti-His antibodies.

We could consistently detect an upper-shifted band of RXRα in GSK-3β dose-dependent manner in various HCC cell lines and HEK293T cells (Fig. 3A and Fig. S3A), which could be specifically erased by calf intestinal alkaline phosphatase (CIP) (Fig. 3B) and inhibited by GSK-3β inhibitors, LiCl, SB415286 and BIO (Fig. 3C and D), indicating that GSK-3β could induce RXRα phosphorylation. Enhanced RXRα phosphorylation was seen in GSK-3β/S9A (mutation with enhanced enzyme activity), while no RXRα phosphorylation event in GSK-3β/K85R (a kinase-dead mutant) (Fig. 3E). Using anti-phosphor-Ser/Thr (p-S/T) antibody, we confirmed that GSK-3β could induce RXRα phosphorylation in HepG2 cells (Fig. 3F) and HCC tumor tissues (Fig. 3G). *In vitro* kinase assays showed that His-GSK-3β could effectively phosphorylate GFP-RXRα protein in the presence of 100 μM ATP (Fig. 3H). Taken together, we demonstrated that RXRα is a direct substrate of GSK-3β.

**Figure 4:**
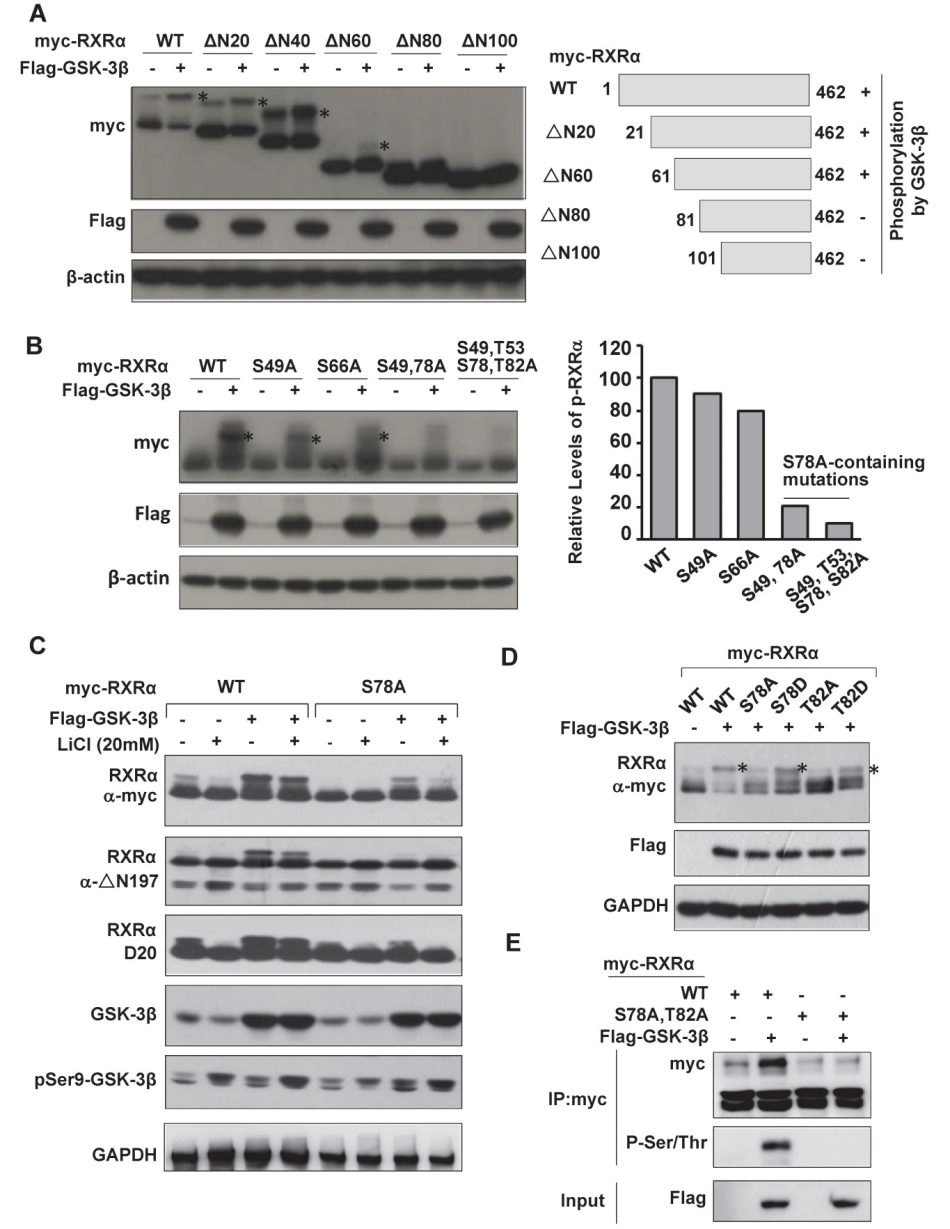
** Identification of RXRα phosphorylation sites by GSK-3β. (A)** HEK293T cells were transfected with Flag-GSK-3β in the presence of myc-RXRα or its various N-terminal truncated forms (ΔN20, ΔN40, ΔN60, ΔN80 and ΔN100). The cells were lysed and blotted with anti-Flag, anti-myc and anti-β-actin antibodies. **(B)** HEK-293T cells were transfected with Flag-GSK-3β in the presence of RXRα or its point mutations including S49A, S66A, S78A, and T82A (alone or in combination). The blots were detected with anti-myc, anti-Flag and anti-β-actin antibodies. **(C)** HepG2 cells were transfected Flag-GSK-3β combined with myc-RXRα or myc-RXRα/S78A. The cells were treated with or without 20 mM LiCl for 3 h. RXRα expression was detected with anti-myc tag or D20 or △N197 antibodies. GSK-3β and its Ser^9^ phosphorylation were examined with anti-Flag and anti-Ser^9^ antibodies. GAPDH expression was used as loading control. **(D)** HepG2 cells were transfected with vector or Flag-GSK-3β in combination with myc-RXRα or its mutants (S78A, S78D, T82A, T82D) for 24 h. The blots were determined with anti-myc, anti-Flag, and anti-GAPDH antibodies. **(E)** HepG2 cells were transfected with Flag-GSK-3β plus myc-RXRα or myc-RXRα/S78A/T82A (AA) for 48 h. The lysates were blotted with anti-myc and anti-p-S/T. The input was detected with anti-Flag antibody. For (A), (B) and (D), RXRα phosphorylation was indicated with *.

We thus proceeded to map GSK-3β-mediated phosphorylation site on RXRα. Deletion mutation analysis showed that GSK-3β could phosphorylate RXRα/ΔN20, ΔN40 and ΔN60, which effect was impaired in ΔN80 and lost in ΔN100, indicating that the putative phosphorylation site is located between 60~100 aa (Fig. 4A). To identify the phosphorylation site, we introduced Ala point mutation into these putative sites. Our results showed that RXRα phosphorylation by GSK-3β was kept at S49A and S66A, but abolished in S78A and S78A-containing mutations (Fig. 4B and C), thus identifying that Ser^78^ is the site for phosphorylation by GSK-3β. Since GSK-3β recognizes sequence motif in the context of S/T-X-X-X-S/T, Thr^82^ was expected as a priming phosphorylation site. We thus also introduced Asp mutation into Ser^78^ (S78D) and Thr^82^ (T82D) to mimic their phosphorylation. As a result, GSK-3β-mediated RXRα phosphorylation was abolished in S78A and T82A, but retained in S78D and T82D (Fig. 4D). The upper-shifted band seen in S78D was due to its acidic carboxylic group-contained aspartic acid, which was unrelated to the activity of GSK-3β 28. Thus, Thr^82^ phosphorylation primes RXRα for subsequent phosphorylation of Ser^78^ by GSK-3β. This phosphorylation event was confirmed with anti-p-S/T antibody (Fig. 4E and data not shown).

### GSK-3β inhibits RXRα transcriptional activity and impairs retinoid signaling

**Figure 5:**
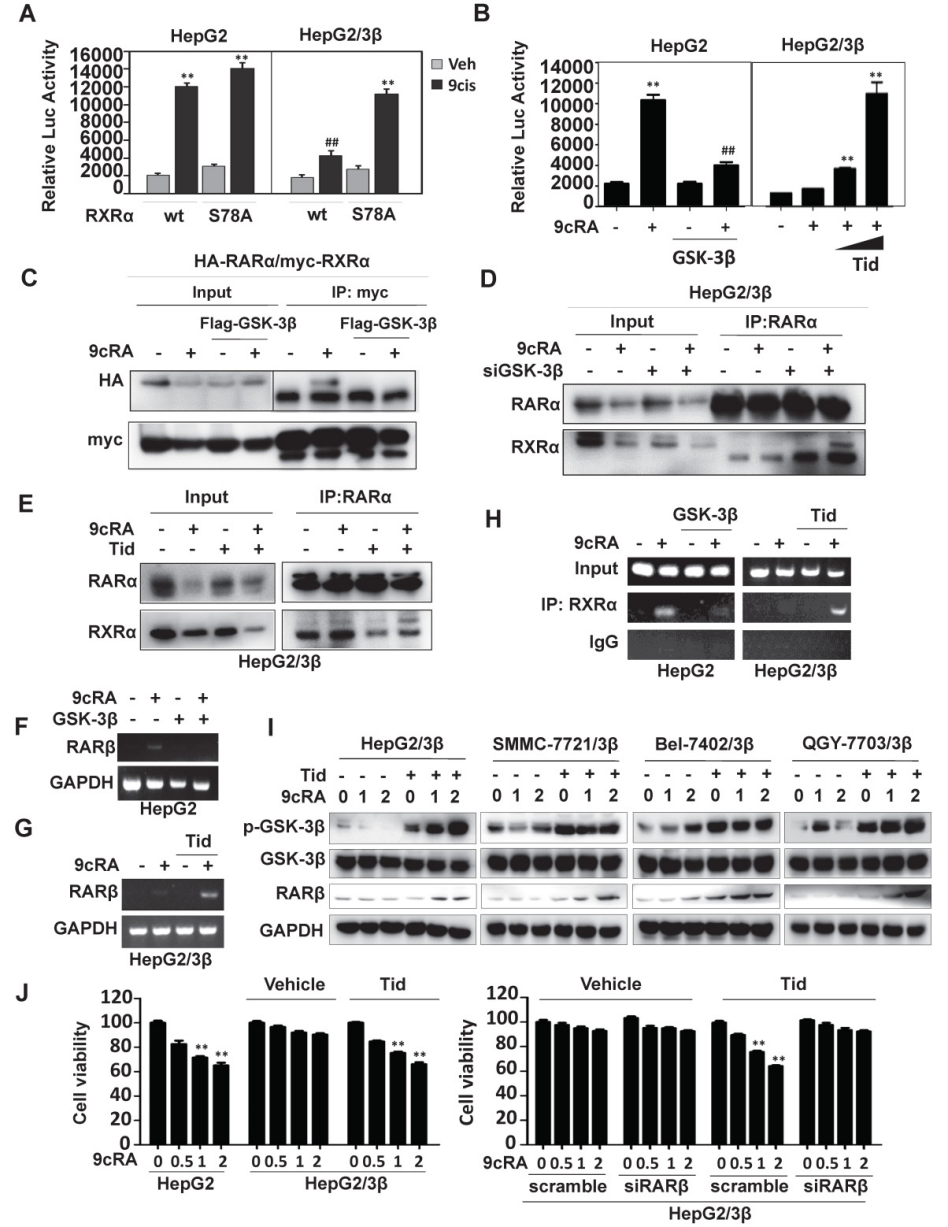
** GSK-3β impairs retinoid signaling. (A)** Reporter assays. HepG2 and HepG2/3β cells were transfected with myc-RXRα or myc-RXRα/S78A. The cells were co-transfected with pGL6-βRARE, Renilla, and HA-RARα for 24 h and then treated vehicle or 1 μM 9-*cis*-RA for 20 h. The fluorescence intensities were determined with Multiskan Spectrum (PerkinElmer). Renilla luciferase activity was used to normalize for transfection efficiency. ^**^p<0.01 (*vs* respective control). **(B)** HepG2 cells were transfected with Flag-GSK-3β or Flag vector, in combination with HA-RARα, myc-RXRα, pGL6-βRARE and Renilla for 24 h. The cells were treated with vehicle or 1μM 9-*cis*-RA for 20 h. HepG2/3β stable cells were transfected with pGL6-βRARE, Renilla, HA-RARα and myc-RXRα. After 24 h transfection, the cells were pretreated with vehicle or with different concentrations of tideglusib (2 μM, 5 μM) for 1 h followed by 1μM 9-*cis*-RA for 20 h. Luciferase activities were similarly detected.^ **^p<0.01 (*vs* respective control); ^##^*p*<0.01 (GSK-3β *vs* mock transfection). **(C)** HepG2 cells were transfected with vector or Flag-GSK-3β in combination with myc-RXRα and HA-RARα for 36 h. The cells were treated with or without 1 μM 9-*cis*-RA for 6 h. The lysates were immunoprecipitated with anti-myc tag and blotted with anti-HA and anti-myc antibodies. **(D)** HepG2/3β cells were transfected with siGSK-3β or scramble (500 pmol in 10 cm dish) for 48 h. The cells were then treated with or without 1 μM 9-*cis*-RA for 6 h. Co-IP was performed with anti-RARα and blotted with anti-RARα and anti-RXRα antibodies. **(E)** HepG2/3β cells were pretreated with 5 μM tideglusib for 1 h and then treated with vehicle or 1 μM 9-*cis*-RA for 6 h. The cell lysates were immunoprecipitated with anti-RARα and blotted with anti-RARα and anti-RXRα (D20) antibodies. For (C), (D), and (E), the inputs were detected with 5% of whole cell lysates. **(F)(G)** RARβ mRNA expression. HepG2 cells were transfected with vector or Flag-GSK-3β for 24 h (F). HepG2/3β cells were pretreated with 5 μM tideglusib for 1 h (G). Both HepG2 and HepG2/3β cells were treated with 1 μM 9-*cis*-RA or vehicle for 24 h. RARβ and GAPDH transcripts were detected with RT-PCR. **(H)** CHIP assays. HepG2 cells were transiently transfected with Flag-GSK-3β, while HepG2/3β stable cells were pretreated with 5 μM tideglusib for 1 h. Both cell lines were treated with vehicle or 1 μM 9-*cis*-RA for 20 h. The Chromatin DNA was purified and immunoprecipitated with anti-RXRα (D20) antibody or nonspecific IgG. The IPs were subjected to RT-PCR analysis by using specific RARβ promoter primers as indicated in Materials and Methods. **(I)** Different GSK-3β stable cell lines were pretreated with 5 μM tideglusib and then treated with vehicle or with increasing concentrations of 9-*cis*-RA for 20 h. The lysates were blotted with anti-RARβ, anti-Flag, anti-Ser^9^ GSK-3β and anti-GAPDH antibodies. **(J)** HepG2 and HepG2/3β cells were pretreated with 5 μM tideglusib and then exposed to vehicle or increasing concentrations of 9-*cis*-RA for 48 h. HepG2/3β cells were also transfected with RARβ siRNA or scramble siRNA and then subjected to similar treatments. The cell proliferation was detected with MTT. ^**^p<0.01 (*vs* respective control).

A luciferase reporter containing βRARE sequence was constructed. This reporter was 9-*cis*-RA inducible in the presence of RXRα, which could be inhibited by overexpression of GSK-3β (Fig. 5A). When Ser^78^ was mutated into Ala^78^ (RXRα/S78A), GSK-3β-mediated βRARE inhibition could be reactivated by 9-*cis*-RA (Fig. 5A). 9-*cis*-RA could also restore βRARE activity *via* GSK-3β inhibition by tideglusib (Fig. 5B). Such phenomenon was reproducible in SMMC7721 and Bel-7402 (Fig. S3B and S3C).

We then investigated whether GSK-3β could modulate the heterodimeric capacity of RXRα. As expected, 9-*cis*-RA could induce RXRα interaction with RARα. When overexpression of GSK-3β, 9-*cis*-RA-induced RXRα:RARα complex was dismantled (Fig. 5C and D). Conversely, the complex could be reassembled when GSK-3β was knocked down by specific siRNA (Fig. 5D) or inactivated by tideglusib (Fig. 5E, Fig. S3D and S3E).

RXRα could also form dimer with itself. We thus also study the effect of GSK-3β on 9-cis-RA-induced TREpal luciferase reporter activity, which expression was driven by RXRα:RXRα. We showed that overexpression of GSK-3β could strongly inhibit the formation of RXRα homodimer, which could be rescued when GSK-β was inactivated by tideglusib (Fig. S3F and S3G). Thus, our results demonstrated that GSK-3β could impair the dimeric capacity and transcriptional activity of RXRα.

RARβ and p21, two direct target genes of RXRα 29, 30, could be induced by 9-*cis*-RA. Such induction was inhibited when overexpression of GSK-3β (Fig. 5F and Fig. S4A). GSK-3β-mediated silence of RARβ and p21 could be relieved when GSK-3β was inactivated by tideglusib or LiCl (Fig. 5G and Fig. S4C). In contrast, 9-*cis*-RA and LiCl alone only played minor role in modulating the expression of p27, Cyclin D1 and Cyclin B1, all of which do not contain RXRα binding sites on their promoters (Fig. S4C). Interestingly, combined treatment of 9-*cis*-RA and LiCl could synergistically induce expression of these genes (Fig. S4C).

RXRα could bind to RARβ and p21 promoters in the presence of 9-*cis*-RA, while overexpression of GSK-3 β inhibited this activity (Fig. 5H and Fig. S4B). 9-*cis*-RA could reactivate RARβ promoter silenced by GSK-3β when combining with tideglusib (Fig. 5H). Consequently, RARβ expression was induced by co-treatment of 9-*cis*-RA and tideglusib (Fig. 5I).

Biologically, overexpression of GSK-3β rendered HCC cells resistant to 9-*cis*-RA (Fig. 5J). When GSK-3β was inactivated by tideglusib, the sensitivity of 9-*cis*-RA was re-established in GSK-3β stably transfected cells. Combination of 9-*cis*-RA and tideglusib effectively induced RARβ expression, which was required for their synergy to inhibit HCC cell growth. Knocking down RARβ largely blocked the synergistic anticancer activity of 9-*cis*-RA/tideglusib (Fig. 5J).

### GSK-3β contributes to sorafenib resistance

**Figure 6:**
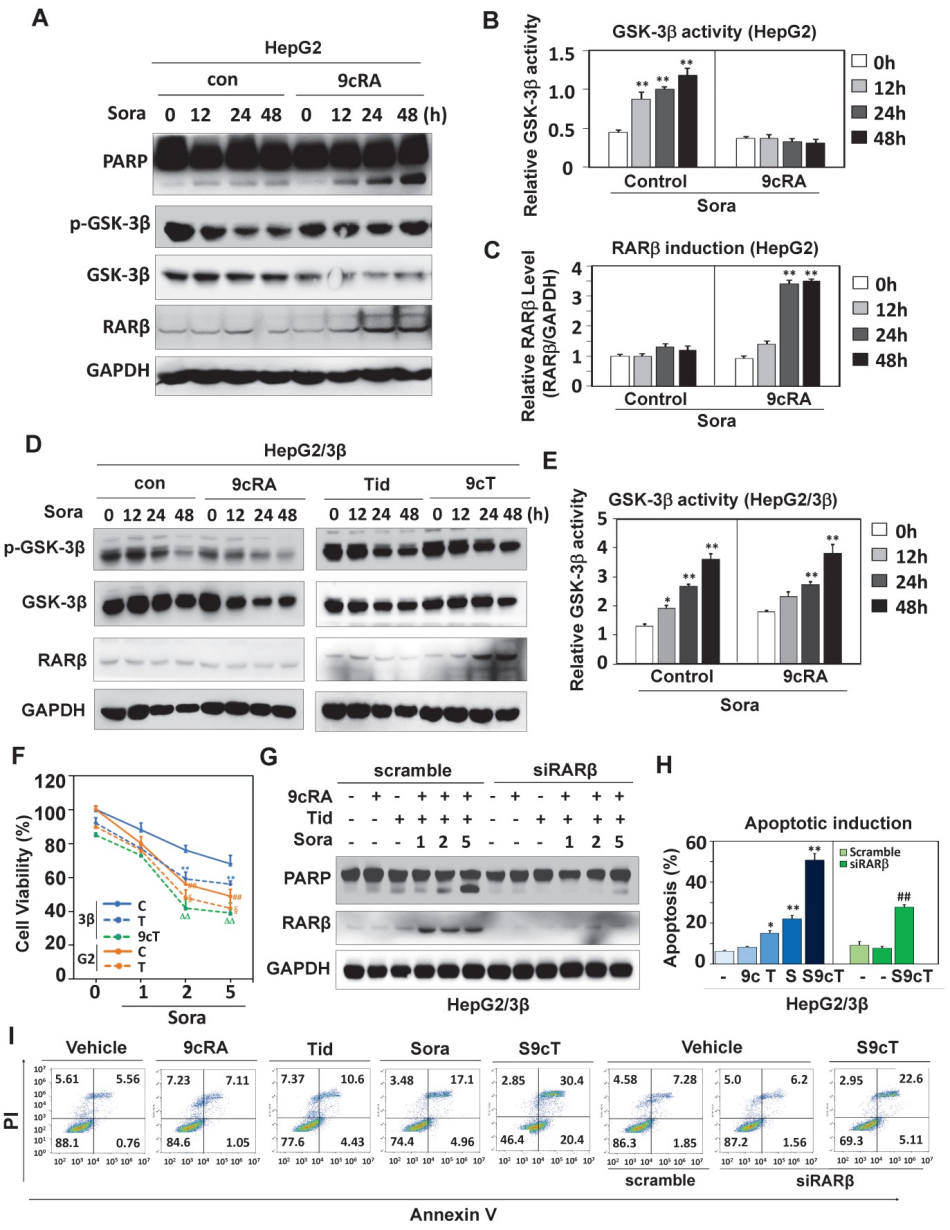
** GSK-3β induces sorafenib resistance. (A)** HepG2 cells were treated with vehicle or 2 μM 9-*cis*-RA in the presence or absence of 5 μM sorafenib for different time intervals. The lysates were blotted with antibodies against PARP, GSK-3β, p-GSK-3β, RARβ and GAPDH. **(B)** GSK-3β activities (GSK-3β/p-GSK-3β) were analysed by normalizing to GAPDH based on band grey values. **(C)** RARβ expression was eemiquantified based on band grey intensities. **(D)** HepG2/3β cells were treated alone or in combination with 2 μM 9-*cis*-RA, 5μM tideglusib and 5 μM sorafenib for different time intervals. Expression levels of GSK-3β, p-GSK-3β, RARβ and GAPDH were determined with respective anti-bodies.** (E)** The role of 9-*cis*-RA on sorafenib-induced GSK-3β activities was analysed based on IB band grey intensities derived in (D). For (B), (C), and (E), ^*^p<0.05, ^**^p<0.01 (*vs* respective controls).** (F)** HepG2/3β cells were treated alone or in combination with 2 μM 9-*cis*-RA, 5μM tideglusib and 5 μM sorafenib for 48 h. The cell growth was examined with MTT assays. ^**^p<0.01 (*vs* control in HepG2/3β), ^##^*p*<0.01 (HepG2/3β *vs* HepG2), **^§^***p*<0.01 (*vs* control in HepG2),**^ΔΔ^***p*<0.01 (*vs* control or tideglusib alone in HepG2/3β).** (G)(H)(I)** HepG2/3β cells were transfected with RARβ siRNA or scramble siRNA. The cells were then treated alone or combined with 2 μM 9-*cis*-RA and 5μM tideglusib in the presence of absence of sorafenib (with increasing concentrations) for 48 h. The lysates were documented using immunoblot with anti-PARP, anti-RARβ and anti-GAPDH antibodies (G) Apoptotic induction was analysed with Flow Cytometry through Annexin V-FITC/PI staining (H). Representative results were provided (I) ^*^p<0.05 and ^**^p<0.01 (*vs* respective control); ^##^*p*<0.01 (RARβ siRNA *vs* scramble). C, control; T, tideglusib; S, sorafenib; S9c, co-treatment with sorafenib and 9-*cis*-RA; S9cT​, combination of sorafenib, 9-*cis*-RA and tideglusib; 9cRA, 9-*cis*-RA.

Sorafenib was shown to activate GSK-3β 31-33. We then decided to study the role of GSK-3β in the action of sorafenib. Although sorafenib could not affect GSK-3β expression, it extensively increased GSK-3β activity (reduced Ser^9^ phosphorylation) in both HepG2 (Fig. 6A and B) and HepG2/3β (Fig. 6D and E). Interestingly, 9-*cis*-RA could reverse sorafenib-induced GSK-3β activation (increased Ser^9^ phosphorylation) in cells with low endogenous GSK-3β (HepG2) (Fig. 6A, 6B and Fig. S5A), but failed to do so in GSK-3β overexpressed cells (HepG2/3β) (Fig. 6D and E). Consistently, 9-*cis*-RA could not affect RXRα phosphorylation when GSK-3β was overexpressed (Fig. S5B). Sorafenib-induced PARP cleavage was enhanced when RARβ was induced by 9-*cis*-RA (Fig. 6A and C).

Biologically, sorafenib resistance was observed in various GSK-3β stable cell lines compared to their vector-transfected counterparts (Fig. 6F and Fig. S6A). Sorafenib response was reestablished in HepG2/3β when GSK-3β activity was inhibited by tideglusib. The enhancement of sorafenib response regarding its anti-proliferation activity (Fig. 6F) and anti-colony formation (Fig. S6B) were achieved by co-treatment of 9-*cis*-RA and tideglusib, which combination could effectively induce RARβ expression and PARP cleavage (Fig. 6G). When RARβ was silenced by specific siRNA, the dose-dependent effect of sorafenib on inducing PARP cleavage was inhibited even in the presence of 9-*cis*-RA/tideglusib (Fig. 6G). We then used flow cytometry to evaluate the synergy of 9-*cis*-RA/tideglusib on enhancing the apoptotic effect of sorafenib. Sorafenib could alone induce 22.1% apoptotic cell death, which effect was promoted to 50.8% by combining with 9-*cis*-RA/tideglusib. The enhancement of 9-*cis*-RA/tideglusib on sorafenib apoptotic response was impaired when silencing RARβ (Fig. 6H, 6I and Fig. S6C). Thus, our results demonstrated that GSK-3β-mediated RARβ inhibition was responsible for sorafenib resistance in HCC cells.

### Targeting GSK-3β enhances the anticancer effect of sorafenib

**Figure 7:**
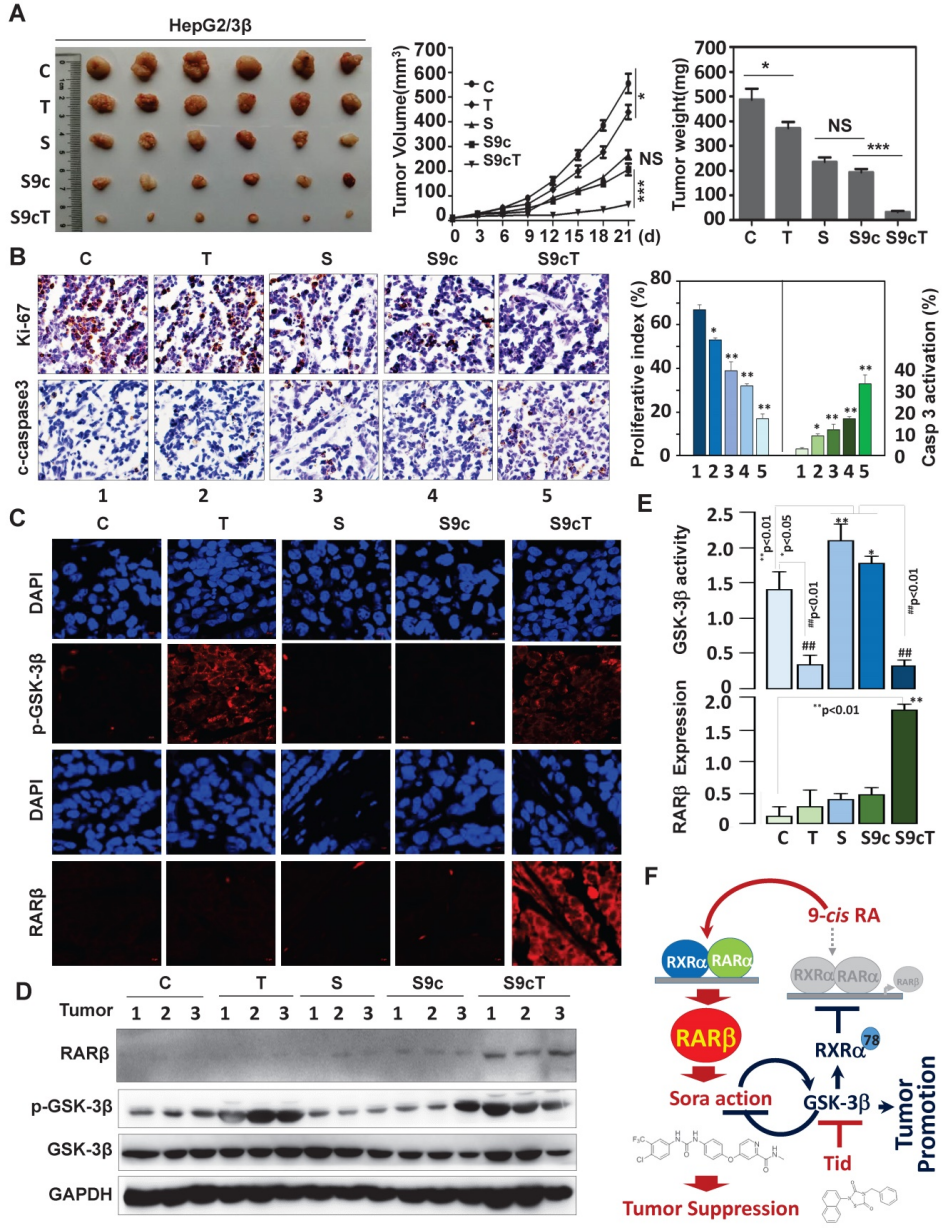
** Animal Experiments. (A)** HepG2/3β cells (2 × 10^6^ cells) were transplanted subcutaneously into mice's posterior flanks. 3 days later, the mice were intraperitoneally injected with 10 mg/kg sorafenib, 2 mg/kg 9-*cis*-RA, and 5 mg/kg tideglusib, alone or combination every other day. Tumors were collected at Day 21. The tumor volume were evaluated every 3 days after treatment. The tumor weight were measured.** (B)** Immunostaining of Ki-67 expression and caspase 3 cleavage were detected with respective antibodies (left panel), which expression were also quantified (right panel). ^*^p<0.05 and ^**^p<0.01 (*vs* respective control). **(C)** Tumor paraffin sections were immunostained with anti-p-GSK-3β and anti-RARβ antibodies and detected with anti-rabbit cy3 conjugated secondary antibodies. DAPI counterstaining was provided. **(D)** Tumors were lysed for IB analysis for detection of RARβ, GSK-3β, p-GSK-3β. GAPDH expression was served as control. **(E)** Quantification of sorafenib-induced GSK-3β activities was analysed as described in Figure 6B.** (F)** Mechanistic scheme. The synergy of 9-*cis*-RA/tideglusib on enhancing the anti-HCC action of sorafenib was summarized. C, control; T, tideglusib; S, sorafenib; 9cRA, 9-cis-RA; S9c, treatment with sorafenib and 9-*cis*-RA; S9cT​, combination of sorafenib, 9-*cis*-RA and tideglusib.

The significance of GSK-3β/RARβ involved in regulation of sorafenib treatment response was finally determined* in vivo*. Targeting GSK-3β could significantly inhibit tumor growth of subcutaneous or orthotopical xenografts (Fig. 7A and Fig. S6D). We found that inactivation of GSK-3β by tideglusib could significantly shrink tumor (22.3% inhibition), inhibit Ki-67 expression (14.2%) and induce caspase 3 activation (9.1%), further supporting that overexpression of GSK-3β might be a tumor promoter in HCC (Fig. 7A and B). Sorafenib treatment resulted in tumor inhibition by 48.3%, which effect could be largely enhanced to 93.4% by combining 9-*cis*-RA/tideglusib (Fig. 7A). Consistently, sorafenib-induced Ki-67 inhibition and caspase 3 activation were greatly promoted by 9-*cis*-RA/tideglusib from 28.4% to 50.2% and from 12.4% to 30.3% respectively (Fig. 7B). 9-*cis*-RA was alone inefficient to improve the anti-tumor effect of sorafenib when GSK-3β remained active (Fig. 7A, B and D). Combination of 9-*cis*-RA/tideglusib/sorafenib did not affect the mouse weight and change the normal histological characteristics of various tissues including liver, lung, kidney, heart, and spleen, demonstrating that this strategy has less toxic side effect (Fig. S7A and B). Mechanistically, tideglusib and 9-*cis*-RA/tideglusib could efficiently inactivate GSK-3β, but only combination could strongly induce RARβ expression (Fig. 7C-E). Interestingly, RARβ expression by 9-*cis*-RA/tideglusib was extensively translocated into cytoplasm (Fig. 7C), implying that the nuclear export of RARβ is responsible for apoptotic induction and tumor growth inhibition. The involvement of GSK-3β/RARβ in sorafenib action was summarized in Fig. 7F.

## Discussion

Functional GSK-3β is recently demonstrated to confer tumor development and poor prognosis in a wide range of solid tumors 34, 35. It is thus widely attempted to design GSK-3β inhibitor for cancer treatment 36-38. However, the concern is its another important function in suppressing tumor growth 2. Inhibition of active GSK-3β, whether beneficially or detrimentally, is highly dependent on contextual environment and clinical settings 35, 39. Disclosing the role and mechanism of GSK-3β in tumor will help develop new therapeutic strategy. HCC is the fourth most common tumor worldwide but with very limited treatment options 24, 40. The expression and therapeutic significance of GSK-3β in HCC remain largely unexplored. Although the samples we examined are small, we could consistently demonstrate that GSK-3β is increased in almost every tumor and upregulation of ≥1.5-fold is seen in 66.7% HCC (Fig. 1A, B and E). Increased GSK-3β is closely associated with shorter overall survival (OS) (Fig. 1E). Consistently, it was recently demonstrated that GSK-3β is overexpressed in HCC and targeting GSK-3β can induce degradation of c-FLIPL, a master anti-apoptotic regulator 25. Our study further showed that overexpression of GSK-3β conferred HCC cell proliferation, colony formation and tumor development, while targeting GSK-3β by tideglusib can significantly induce about 22.3% of growth inhibition in HepG2/3β xenografts (Fig. 7A). Thus, we demonstrated that overexpression of functional GSK-3β supports HCC growth. Since overexpression of GSK-3β renders HCC resistant to certain chemotherapies like retinoid and sorafenib, the therapeutic significance of targeting GSK-3β may lie on its combination with other anticancer drugs.

Hepatocarcinogenesis is closely linked to impaired retinoid metabolism and altered retinoid receptors 15, 41, 42. GSK-3β is recently suggested to be a modulator of retinoid signaling as it strongly inhibits RARα-dependent myeloid leukemia differentiation in response to* all-trans* retinoic acid treatment 16, 17. However, the roles of retinoid receptors in leukemia and solid tumors can be quite different. The therapeutic effects of retinoids are usually less efficacy in solid tumors than leukemia. The mechanism and implication of GSK-3β-mediated impairment of retinoid signaling in solid tumors have not been reported. We demonstrated here that overexpression of GSK-3β can inhibit RARβ expression and impair retinoid signaling in HCC (Fig. 1 and 5). RARβ expression is required for mediating retinoid action 30, but this protein is frequently down-regulated in HCC with poorly understood mechanism 43. It was demonstrated that chromatin hypermethylation can impact negatively on RARβ expression 44. Interestingly, GSK-3β was shown to play a fundamental role in maintaining DNA methylation 45. There are currently no reports on GSK-3β regulation of RARβ. We demonstrated here that GSK-3β-mediated RARβ inhibition is attributed to its direct inactivation of RXRα (Fig. 5D), suggesting that a functional RXRα is required for RARβ induction. We identified RXRα as a new substrate for GSK-3β. GSK-3β can directly interact with and phosphorylate RXRα at Ser^78^ within its N-terminal proline-directed context of S/T-X-X-X-S/T (Fig. 4). Such modification renders RXRα incapable of heterodimerizing with RARα to activate retinoic acid response element on RARβ promoter (Fig. 5). Targeting GSK-3β can recover the function of RXRα (Fig. 5D) and promote retinoid-induced RARβ expression *in vitro* (Fig. 5G) and *in vivo* (Fig. 7D). Our results thus disclosed a novel mechanism by which GSK-3β regulates RARβ expression in HCC.

Deregulation of RARβ-mediated retinoid signaling by GSK-3β may at least partially explain why clinical trials of some classical retinoids like β-retinoic acid have no proven benefit in HCC 46. Interestingly, clinical trial of acyclic retinoid, a synthetic analog of retinoids that target at phosphorylated RXRα, revealed a promising effect in reducing the incidence rate of secondary HCC by about 20% 47, 48.

Sorafenib, a multi-kinase targeted anti-cancer drug, is being widely used to treat HCC 19, 20 but with significant treatment resistance. We thus asked if GSK-3β-mediated RARβ inhibition could impact on sorafenib treatment response. We found that sorafenib can extensively activate GSK-3β both *in vitro* (Fig. 6A-D) 32 and in tumor microenvironment (Fig. 7E). Since GSK-3β is highly expressed in HCC, sorafenib treatment will generate abundantly hyperactive GSK-3β. Overexpression of functional GSK-3β strongly inhibits sorafenib action as indicated in various GSK-3β stable liver cell lines *vs* their vector-transfected counterparts (Fig. 6F and Fig. S6A). 9-*cis*-RA cannot alone induce RARβ expression in GSK-3β stable cell lines, in which RARβ is silenced by GSK-3β. Targeting GSK-3β by tideglusib can greatly potentiate 9-*cis*-RA activation of RARβ-dependent signaling. Importantly, reactivation of RARβ-dependent signaling that is inhibited by overexpression of GSK-3β returns profoundly unexpected sorafenib treatment outcome (tumor inhibition raised sharply from 48.3% to 93.4%) (Fig. 7A). In this study, we only used low dose of tideglusib in animal experiment by considering that GSK-3β is normally a critical regulator of cell metabolism and homeostasis. In addition, tideglusib has been demonstrated to have fewer side effects under phase II trial in Alzheimer's disease treatment 49, 50. On the other hand, normal tissues are resistant to 9-cis-RA-induced cytoxicity 51. Combination of tideglusib and 9-*cis*-RA do not exacerbate deleterious effect of sorafenib in liver and other normal tissues (Fig. S7B).

In summary, our findings suggest that HCC may take advantage of GSK-3β overexpression to support its growth possibly through interfering RARβ-mediated retinoid signalling. The discovery of GSK-3β/RARβ in sorafenib treatment response may help design improved strategy to overcome the significant treatment resistant problem of sorafenib in clinic.

## Supplementary Material

Supplementary figures and table.

## Abbreviations

HCC: hepatocellular carcinoma****
GSK-3: Glycogen Synthase Kinase-3
RAR: retinoic acid receptor
RXR: retinoid X receptor
RARE: retinoic acid response element
TREpal: thyroid hormone response element
9cRA: 9-*cis*-RA
CIP: calf intestinal alkaline phosphatase
RT-PCR: reverse transcription-polymerase chain reaction
siRNA: small interfering RNA
MTT: 3-(4,5-Dimethyl-2-thiazolyl)-2,5-diphenyl-2H-tetrazolium bromide
DAPI: 4,6-Diamidino-2-phenylindole
IPTG: isopropyl-1-thio-b-D-galactopyranoside
Co-IP: co-immunoprecipitation
CHIP: chromatin immunoprecipitation assay
GEPIA: gene expression profiling interactive analysis
OS: overall survival

## References

[1] Nagini S, Sophia J, Mishra R (2019). Glycogen synthase kinases: Moonlighting proteins with theranostic potential in cancer. Semin Cancer Biol.

[2] Beurel E, Grieco SF, Jope RS (2015). Glycogen synthase kinase-3 (GSK3): regulation, actions, and diseases. Pharmacol Ther.

[3] Patel S, Woodgett J (2008). Glycogen synthase kinase-3 and cancer: good cop, bad cop?. Cancer Cell.

[4] Doble BW, Woodgett JR (2003). GSK-3: tricks of the trade for a multi-tasking kinase. J Cell Sci.

[5] Shakoori A, Ougolkov A, Yu ZW, Zhang B, Modarressi MH, Billadeau DD (2005). Deregulated GSK3beta activity in colorectal cancer: its association with tumor cell survival and proliferation. Biochem Biophys Res Commun.

[6] Wang HL, Hart J, Fan L, Mustafi R, Bissonnette M (2011). Upregulation of glycogen synthase kinase 3beta in human colorectal adenocarcinomas correlates with accumulation of CTNNB1. Clin Colorectal Cancer.

[7] Luo J (2009). Glycogen synthase kinase 3beta (GSK3beta) in tumorigenesis and cancer chemotherapy. Cancer Lett.

[8] Ma C, Wang J, Gao Y, Gao TW, Chen G, Bower KA (2007). The role of glycogen synthase kinase 3beta in the transformation of epidermal cells. Cancer Res.

[9] Yin Y, Kizer NT, Thaker PH, Chiappinelli KB, Trinkaus KM, Goodfellow PJ (2013). Glycogen synthase kinase 3beta inhibition as a therapeutic approach in the treatment of endometrial cancer. Int J Mol Sci.

[10] Paz H, Pathak N, Yang J (2014). Invading one step at a time: the role of invadopodia in tumor metastasis. Oncogene.

[11] Gaisina IN, Gallier F, Ougolkov AV, Kim KH, Kurome T, Guo S (2009). From a natural product lead to the identification of potent and selective benzofuran-3-yl-(indol-3-yl)maleimides as glycogen synthase kinase 3beta inhibitors that suppress proliferation and survival of pancreatic cancer cells. J Med Chem.

[12] Hilliard TS, Gaisina IN, Muehlbauer AG, Gaisin AM, Gallier F, Burdette JE (2011). Glycogen synthase kinase 3beta inhibitors induce apoptosis in ovarian cancer cells and inhibit in-vivo tumor growth. Anticancer Drugs.

[13] di Masi A, Leboffe L, De Marinis E, Pagano F, Cicconi L, Rochette-Egly C (2015). Retinoic acid receptors: from molecular mechanisms to cancer therapy. Mol Aspects Med.

[14] Cortes E, Lachowski D, Rice A, Chronopoulos A, Robinson B, Thorpe S (2019). Retinoic Acid Receptor-beta Is Downregulated in Hepatocellular Carcinoma and Cirrhosis and Its Expression Inhibits Myosin-Driven Activation and Durotaxis in Hepatic Stellate Cells. Hepatology.

[15] Yan TD, Wu H, Zhang HP, Lu N, Ye P, Yu FH (2010). Oncogenic potential of retinoic acid receptor-gamma in hepatocellular carcinoma. Cancer Res.

[16] Si J, Mueller L, Collins SJ (2011). GSK3 inhibitors enhance retinoic acid receptor activity and induce the differentiation of retinoic acid-sensitive myeloid leukemia cells. Leukemia.

[17] Gupta K, Gulen F, Sun L, Aguilera R, Chakrabarti A, Kiselar J (2012). GSK3 is a regulator of RAR-mediated differentiation. Leukemia.

[18] Gao W, Liu J, Hu M, Huang M, Cai S, Zeng Z (2013). Regulation of proteolytic cleavage of retinoid X receptor-alpha by GSK-3beta. Carcinogenesis.

[19] Cheng AL, Kang YK, Chen Z, Tsao CJ, Qin S, Kim JS (2009). Efficacy and safety of sorafenib in patients in the Asia-Pacific region with advanced hepatocellular carcinoma: a phase III randomised, double-blind, placebo-controlled trial. Lancet Oncol.

[20] Llovet JM, Ricci S, Mazzaferro V, Hilgard P, Gane E, Blanc JF (2008). Sorafenib in advanced hepatocellular carcinoma. N Engl J Med.

[21] Niu L, Liu L, Yang S, Ren J, Lai PBS, Chen GG (2017). New insights into sorafenib resistance in hepatocellular carcinoma: Responsible mechanisms and promising strategies. Biochim Biophys Acta Rev Cancer.

[22] Berasain C (2013). Hepatocellular carcinoma and sorafenib: too many resistance mechanisms?. Gut.

[23] Cong WM, Bu H, Chen J, Dong H, Zhu YY, Feng LH (2016). Practice guidelines for the pathological diagnosis of primary liver cancer: 2015 update. World J Gastroenterol.

[24] Li L, Wang H (2016). Heterogeneity of liver cancer and personalized therapy. Cancer Lett.

[25] Zhang N, Liu X, Liu L, Deng Z, Zeng Q, Pang W (2018). Glycogen synthase kinase-3beta inhibition promotes lysosome-dependent degradation of c-FLIPL in hepatocellular carcinoma. Cell Death Dis.

[26] Iwagami Y, Huang CK, Olsen MJ, Thomas JM, Jang G, Kim M (2016). Aspartate beta-hydroxylase modulates cellular senescence through glycogen synthase kinase 3beta in hepatocellular carcinoma. Hepatology.

[27] de The H, Vivanco-Ruiz MM, Tiollais P, Stunnenberg H, Dejean A (1990). Identification of a retinoic acid responsive element in the retinoic acid receptor beta gene. Nature.

[28] Zhou A, Lin K, Zhang S, Chen Y, Zhang N, Xue J (2016). Nuclear GSK3beta promotes tumorigenesis by phosphorylating KDM1A and inducing its deubiquitylation by USP22. Nat Cell Biol.

[29] Tanaka T, Suh KS, Lo AM, De Luca LM (2007). p21WAF1/CIP1 is a common transcriptional target of retinoid receptors: pleiotropic regulatory mechanism through retinoic acid receptor (RAR)/retinoid X receptor (RXR) heterodimer and RXR/RXR homodimer. J Biol Chem.

[30] Tang XH, Gudas LJ (2011). Retinoids, retinoic acid receptors, and cancer. Annu Rev Pathol.

[31] Panka DJ, Cho DC, Atkins MB, Mier JW (2008). GSK-3beta inhibition enhances sorafenib-induced apoptosis in melanoma cell lines. J Biol Chem.

[32] Dudgeon C, Peng R, Wang P, Sebastiani A, Yu J, Zhang L (2012). Inhibiting oncogenic signaling by sorafenib activates PUMA via GSK3beta and NF-kappaB to suppress tumor cell growth. Oncogene.

[33] Kawazoe H, Bilim VN, Ugolkov AV, Yuuki K, Naito S, Nagaoka A (2012). GSK-3 inhibition in vitro and in vivo enhances antitumor effect of sorafenib in renal cell carcinoma (RCC). Biochem Biophys Res Commun.

[34] Cervello M, Augello G, Cusimano A, Emma MR, Balasus D, Azzolina A (2017). Pivotal roles of glycogen synthase-3 in hepatocellular carcinoma. Adv Biol Regul.

[35] Takahashi-Yanaga F (2013). Activator or inhibitor? GSK-3 as a new drug target. Biochem Pharmacol.

[36] Saraswati AP, Ali Hussaini SM, Krishna NH, Babu BN, Kamal A (2018). Glycogen synthase kinase-3 and its inhibitors: Potential target for various therapeutic conditions. Eur J Med Chem.

[37] Mathuram TL, Reece LM, Cherian KM (2018). GSK-3 Inhibitors: A Double-Edged Sword? - An Update on Tideglusib. Drug Res (Stuttg).

[38] Walz A, Ugolkov A, Chandra S, Kozikowski A, Carneiro BA, O'Halloran TV (2017). Molecular Pathways: Revisiting Glycogen Synthase Kinase-3beta as a Target for the Treatment of Cancer. Clin Cancer Res.

[39] McCubrey JA, Davis NM, Abrams SL, Montalto G, Cervello M, Basecke J (2014). Diverse roles of GSK-3: tumor promoter-tumor suppressor, target in cancer therapy. Adv Biol Regul.

[40] Villanueva A (2019). Hepatocellular Carcinoma. N Engl J Med.

[41] Zeng W, Zhang C, Cheng H, Wu YL, Liu J, Chen Z (2017). Targeting to the non-genomic activity of retinoic acid receptor-gamma by acacetin in hepatocellular carcinoma. Sci Rep.

[42] Wang PY, Zeng WJ, Liu J, Wu YL, Ma Y, Zeng Z (2017). TRC4, an improved triptolide derivative, specifically targets to truncated form of retinoid X receptor-alpha in cancer cells. Biochem Pharmacol.

[43] Sever CE, Locker J (1991). Expression of retinoic acid alpha and beta receptor genes in liver and hepatocellular carcinoma. Mol Carcinog.

[44] Moison C, Senamaud-Beaufort C, Fourriere L, Champion C, Ceccaldi A, Lacomme S (2013). DNA methylation associated with polycomb repression in retinoic acid receptor beta silencing. FASEB J.

[45] Meredith GD, D'Ippolito A, Dudas M, Zeidner LC, Hostetter L, Faulds K (2015). Glycogen synthase kinase-3 (Gsk-3) plays a fundamental role in maintaining DNA methylation at imprinted loci in mouse embryonic stem cells. Mol Biol Cell.

[46] Meyskens FL Jr, Jacobson J, Nguyen B, Weiss GR, Gandara DR, MacDonald JS (1998). Phase II trial of oral beta-all trans-retinoic acid in hepatocellular carcinoma (SWOG 9157). Invest New Drugs.

[47] Shimizu M, Sakai H, Moriwaki H (2011). Chemoprevention of hepatocellular carcinoma by acyclic retinoid. Front Biosci (Landmark Ed).

[48] Muto Y, Moriwaki H, Saito A (1999). Prevention of second primary tumors by an acyclic retinoid in patients with hepatocellular carcinoma. N Engl J Med.

[49] Lovestone S, Boada M, Dubois B, Hull M, Rinne JO, Huppertz HJ (2015). A phase II trial of tideglusib in Alzheimer's disease. J Alzheimers Dis.

[50] Bharathy N, Svalina MN, Settelmeyer TP, Cleary MM, Berlow NE, Airhart SD (2017). Preclinical testing of the glycogen synthase kinase-3beta inhibitor tideglusib for rhabdomyosarcoma. Oncotarget.

[51] Tatebe H, Shimizu M, Shirakami Y, Tsurumi H, Moriwaki H (2008). Synergistic growth inhibition by 9-cis-retinoic acid plus trastuzumab in human hepatocellular carcinoma cells. Clin Cancer Res.

