# Supplementary material for: The Roles of GSK-3β in Regulation of Retinoid Signaling and Sorafenib Treatment Response in Hepatocellular Carcinoma
Source: Theranostics. 2020 Jan 1;10(3):1230–44. doi: 10.7150/thno.38711 (PMC6956800; doi:10.7150/thno.38711)
Supplement: Supplementary file 1 — Supplementary figures and table. [file thnov10p1230s1.pdf]

Supplementary Data

Table S1. Clinical data and GSK-3β expression of HCC cases

**Patient**	**Age/Gender**	**TNM/stage**	**Tumor Size**	**Site of tumor**	**GSK-3β** (T/N)
1	61/M	$T_3N_1M_0$/IV	7*5*3.5cm	L	↑↑
2	43/M	$T_4N_1M_0$/IV	11*5*2cm	R	↑↑
3	44/M	$T_2N_0M_0$/I-II	3.5*3*2.5cm	R	
4	54/M	$T_2N_0M_0$/I-II	4.5*3.5*3cm	R	
5	34/M	$T_2N_0M_0$/II	3.4*3.2*2cm	R	
6	43/M	$T_2N_0M_0$/II	5*4*4cm	R	↑↑
7	42/M	$T_2N_0M_0$/II	4*3.8*3.5cm	R	↑
8	63/F	$T_2N_0M_0$/II	4*3*3cm	R	↑↑
9	46//F	$T_2N_0M_0$/I-II	3.8*3.5*3cm	L	↑↑
10	63/M	$T_2N_0M_0$/II	3.5*3*3cm	R	
11	53/M	$T_2N_0M_0$/II	3.2*3*3cm	L	↑
12	52/M	$T_2N_0M_0$/II	5.6*5*4cm	R	↑↑
13	42/M	$T_3N_0M_0$/II-III	8*5*5cm	R	↑↑
14	48/F	$T_2N_0M_0$/II	4.5*4*4cm	L	
15	45/M	$T_3N_1M_0$/IV	6.5*6*5cm	R	↑
16	58/M	$T_2N_0M_0$/I-II	4*4*3cm	R	
17	46/M	$T_2N_0M_0$/II	5*4.6*3cm	R	↑
18	48/M	$T_3N_1M_0$/IV	6.5*5*4cm	R	↑↑

Notes: Patients were pathologically diagnosed and staged according to AJCC TNM staging criteria. The patients with remote metastasis or other complicated diseases like diabetes were excluded. All cases recruited were not received any treatment before surgery. M: male; F: female; R: right lobe of liver; L: left lobe of liver; ↑: T/N>1.5; ↑↑: T/N>2.0.

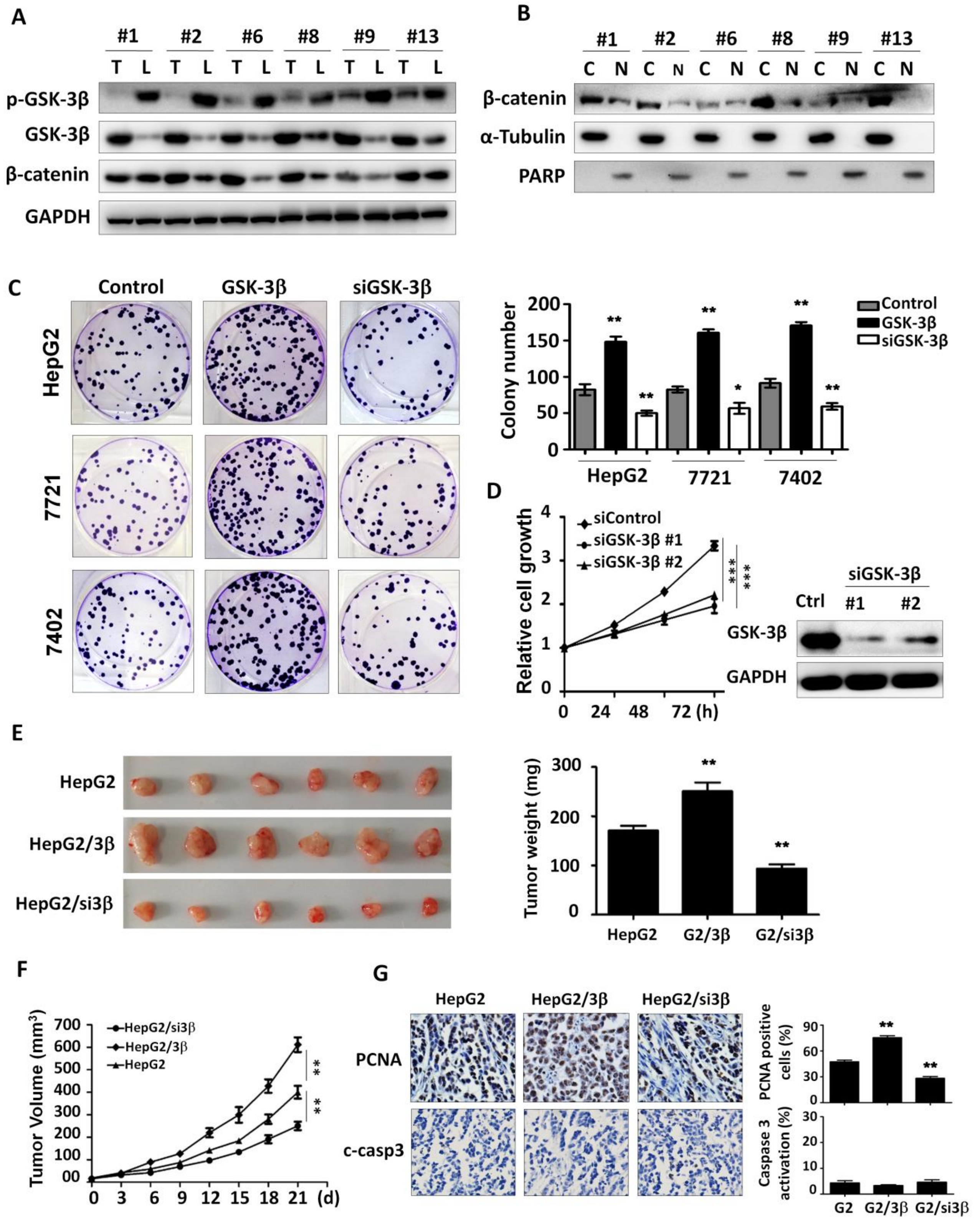

Figure S1. Oncogenic potential of GSK-3β in HCC. (A) Tumor-associated GSK-3β does not correlate with β-catenin down regulation. β-catenin expression was determined by western blotting in those cases with high GSK-3β. **(B)** β-catenin nuclear translocation was examined in tumor tissues with subcellular fractionation. C: cytoplasm; N: nuclear. **(C)** GSK-3β facilitates HCC cell colony formation. Various HCC cells and their stable lines harboured with or knocked down GSK-3β were

grown in 6-well plates for two weeks. The number of foci containing >50 cells was counted and statistically analysed. $^{*}p<0.05$, $^{**}p<0.01$ (*vs* control). **(D)** GSK-3β contributes to HCC cell proliferation. GSK-3β was stably knocked down by specific siRNA in HepG2. Scramble siRNA was served as control. Two HepG2/siβ lines were used. The cells were grown in 96-well plates for different time intervals and subjected to MTT assay. **(E)** GSK-3β confers HCC tumor growth. HepG2, HepG2/3β and HepG2/siβ cells (2×10^6 cells) were transplanted subcutaneously into mice's posterior flanks. After three weeks, the mice were sacrificed. Tumors were collected, weighed and analysed. $^{**}p<0.01$ (*vs* control). **(F)** Tumor volume were recorded every 3 days and analysed. $^{**}p<0.01$ (*vs* control). **(G)** PCNA and c-caspase3 expression were stained. The proliferative index (PCNA positive cells) and caspase 3 activation were statistically analysed. $^{**}p<0.01$ (*vs* control).

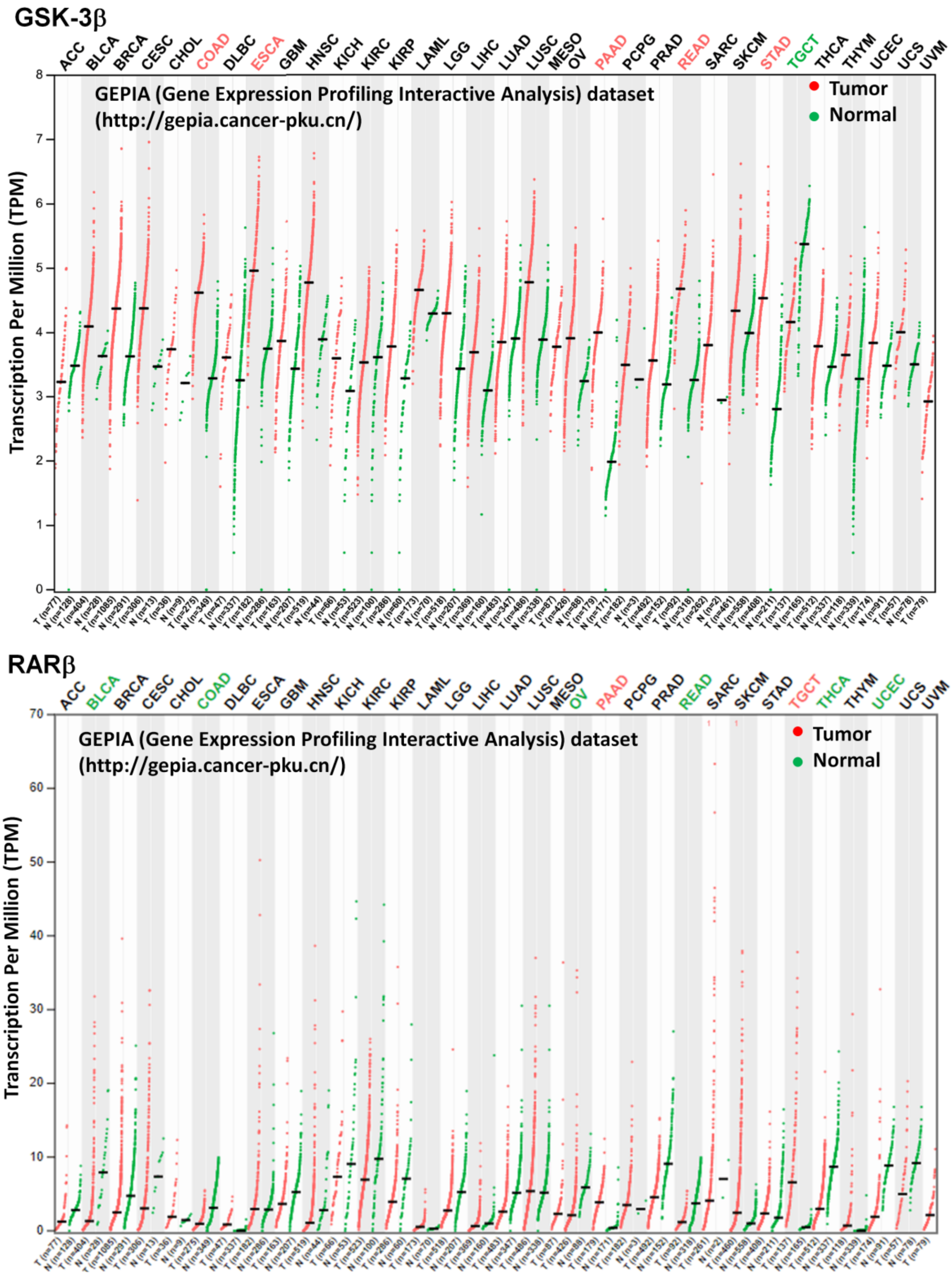

Figure S2. GSK-3β and RARβ expression profiles. The relative expression levels of both genes in tumors (T) and normal (N) tissues of different organs were analysed using the web-based tool GEPIA dataset (Gene Expression Profiling Interactive Analysis, http://gepia.cancer-pku.cn/). Abbreviations: ACC (Adrenocortical carcinoma) BLCA (Bladder Urothelial Carcinoma) BRCA (Breast invasive carcinoma) CESC (Cervical squamous cell carcinoma and endocervical adenocarcinoma) CHOL (Cholangio carcinoma) COAD (Colon adenocarcinoma) DLBC (Lymphoid Neoplasm Diffuse Large B-cell Lymphoma) ESCA (Esophageal carcinoma) GBM (Glioblastoma multiforme) HNSC (Head and Neck squamous cell carcinoma) KICH (Kidney Chromophobe) KIRC (Kidney renal clear cell

carcinoma) KIRP (Kidney renal papillary cell carcinoma) LAML (Acute Myeloid Leukemia) LGG (Brain Lower Grade Glioma) LIHC (Liver hepatocellular carcinoma) LUAD (Lung adenocarcinoma) LUSC (Lung squamous cell carcinoma) MESO (Mesothelioma) OV (Ovarian serous cystadenocarcinoma) PAAD (Pancreatic adenocarcinoma) PCPG (Pheochromocytoma and Paraganglioma) PRAD (Prostate adenocarcinoma) READ (Rectum adenocarcinoma) SARC (Sarcoma) SKCM (Skin Cutaneous Melanoma) STAD (Stomach adenocarcinoma) TGCT (Testicular Germ Cell Tumors) THCA (Thyroid carcinoma) THYM (Thymoma) UCEC (Uterine Corpus Endometrial Carcinoma) UCS (Uterine Carcinosarcoma) UVM (Uveal Melanoma).

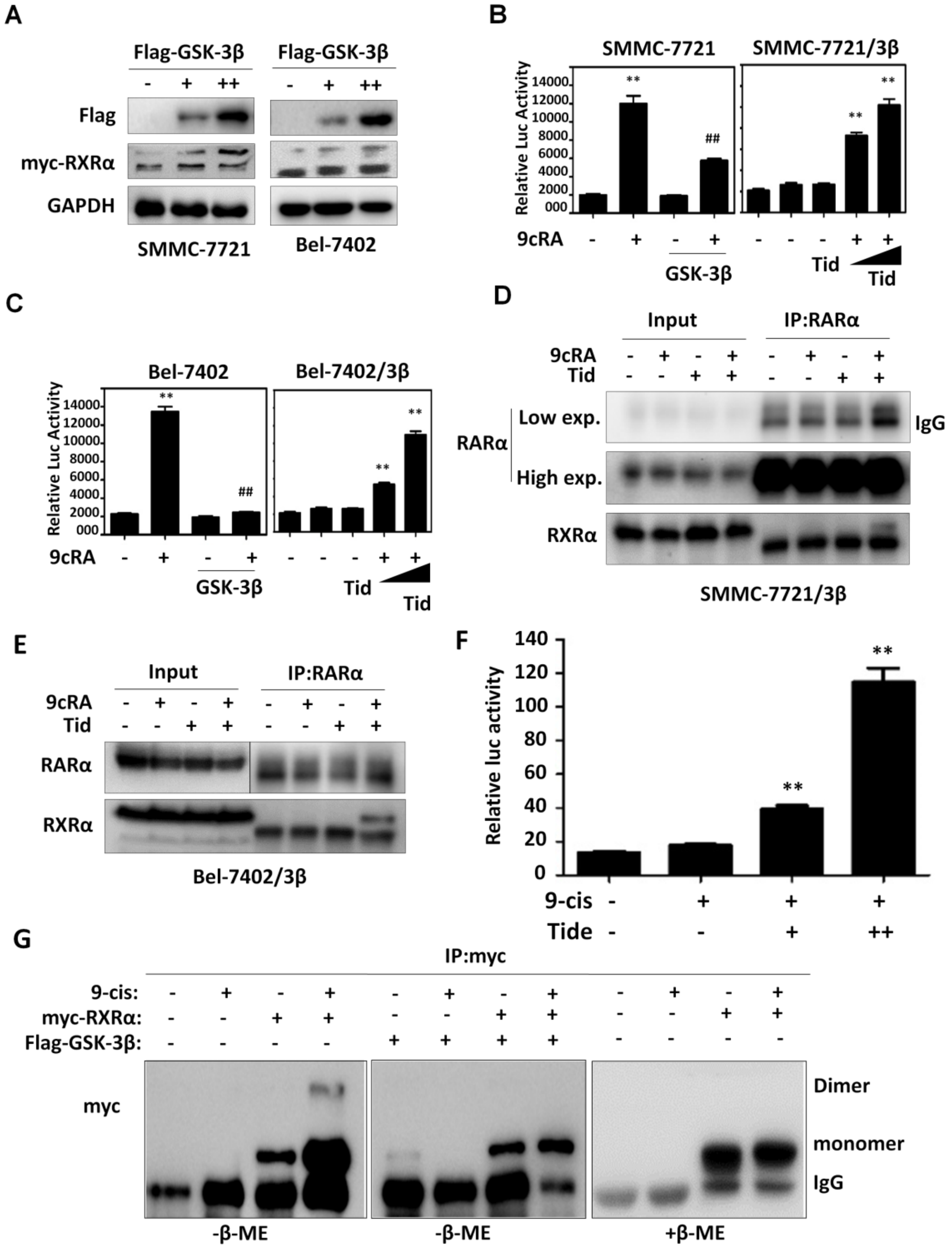

Figure S3. Role of GSK-3β in RXRα function. (A) SMMC-7721 and Bel-7402 cells were transfected with myc-RXRα together with or without increasing concentrations of Flag-GSK-3β. After 36 h of transfection, the cells were lysed and subjected to IB analysis with anti-myc, Flag, and GAPDH antibodies. **(B)(C)** SMMC-7721 and Bel-7402 cells were transfected with Flag-GSK3β or Flag vector, in combination with HA-RARα, myc-RXRα, pGL6-βRARE and Renilla for 24 h and then treated with vehicle or 1 μM 9-*cis*-RA for 20 h. SMMC-7721/3β and Bel-7402/3β stable cells were transfected with pGL6-βRARE, Renilla, HA-RARα and myc-RXRα. After 24 h transfection, the cells were pretreated with vehicle or with different concentrations of tideglusib for 1 h and then

treated with 1 μM 9-*cis*-RA for 20 h. Luciferase activities were similarly determined. $^{**}p<0.01$ (*vs* respective controls); $^{\#\#}p<0.01$ (GSK-3β *vs* mock vector). **(D)(E)** SMMC-7721/3β and Bel-7402/3β cells were pretreated with 5 μM tideglusib for 1 h and then treated with vehicle or 1 μM 9-*cis*-RA for 6 h. The cell lysates were immunoprecipitated with anti-RARα and blotted with anti-RARα and anti-RXRα (D20) antibodies. The inputs were detected with 5% of whole cell lysates. (**F**) Reporter assays. HepG2 cells were co-transfected with myc-RXRα, pGL6-TREpal and Renilla for 24 h. The cells were then subjected to 1 μM 9-*cis*-RA treatment with or without increasing concentrations of tideglusib for 20 h. The fluorescence intensities were determined with Multiskan Spectrum (PerkinElmer). Renilla luciferase activity was used to normalize for transfection efficiency. **(G)** GSK-3β impairs RXRα dimeric capacity. HepG2 cells were transfected with myc-RXRα and Flag-GSK-3β (alone or in combination) for 24 h and then exposed to 9-*cis*-RA for 6 h. Cell lysates were immunoprecipitated by anti-myc tag antibody or IgG in the presence or absence of 1 mM β-Mercapto-Ethanol (β-ME). The IP products were separated by 8% non-denaturing PAGE and blotted with anti-myc tag antibody. Monomer and dimer were indicated.

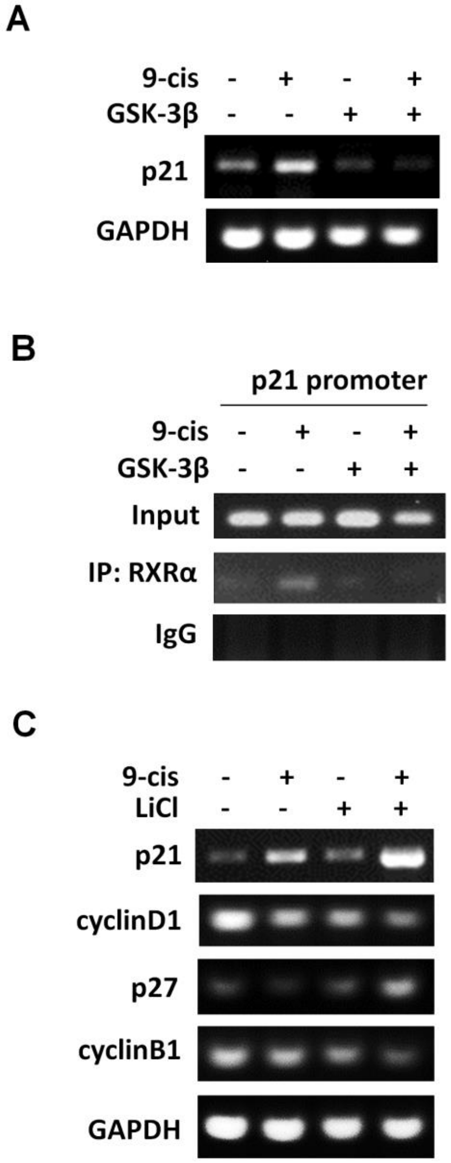

Figure S4. GSK-3β regulation of various genes in response to 9-*cis*-RA. (**A**) HepG2 cells were transfected with vector or Flag-GSK-3β for 24 h. The cell lysates were analysed for p21 and GAPDH transcripts by RT-PCR. (**B**) CHIP assays. HepG2 cells were transfected with Flag-GSK-3β or Flag vector for 24 h. The cells were treated with vehicle or 1 μM 9-*cis*-RA for 20 h. Chromatin DNA was purified and immunoprecipitated with anti-RXRα (D20) antibody or nonspecific IgG. The IPs were subjected to RT-PCR analysis by using specific p21 promoter primers (Forward: 5'-CAGATGAACAATCCATCCTCTG-3'; Reverse: 5'-TGCCAAATAACCCTCATTTGC-3'). The input was detected with the same primers of p21 promoter. **(C)** HepG2 cells were treated with 1 μM 9-*cis*-RA in the presence or absence of 20 mM LiCl for 20 h. The transcripts of p21, p27, cylcin B1, cyclin D1 and GAPDH were analysed with RT-PCR. The primer sequences included: p21, 5'-CCC AAG CTC TAC CTT CCC AC-3' (Forward); 5'-CTG AGA GTC TCC AGG TCC AC-3' (Reverse); p27, 5'-GCA AGT ACG AGT GGC AAG AG-3' (Forward); 5'-GTC GCT TCC TTA TTC CTG CG-3' (Reverse); cyclin B1, 5'-TGA GGA AGA GCA AGC AGT CA-3' (Forward); 5'-AAC ATG GCA GTG ACA CCA AC-3' (Reverse); cyclin D1, 5'-GCA TGT TCG TGG CCT CTA AG-3' (Forward); 5'-CGT GTT TGC GGA TGA TCT GT-3' (Reverse).

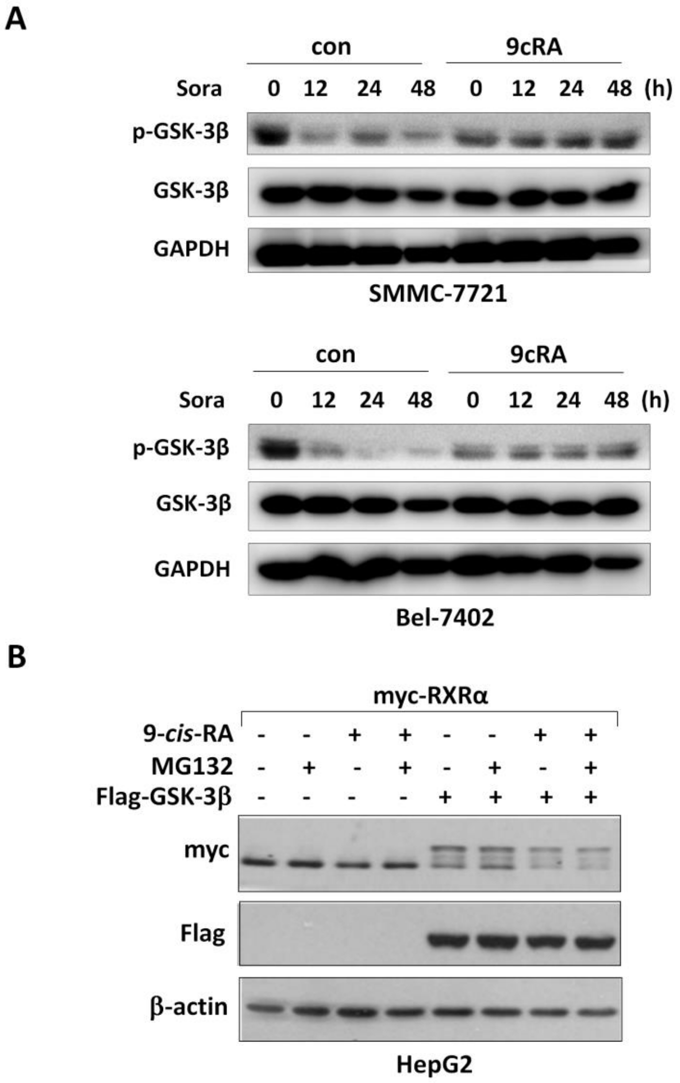

Figure S5. 9-*cis*-RA modulates GSK-3β activity. (A) SMMC-7721 and Bel-7402 cells were treated with vehicle or 2 μM 9-*cis*-RA in the absence or presence of 5 μM sorafenib for different time intervals. The lysates were blotted with antibodies against GSK-3β, p-GSK-3β, and GAPDH. **(B)** HepG2 cells were transfected with myc-RXRα alone or co-transfected with Flag-GSK-3β for 24 h. The cells were treated with 1 μM 9cRA with or without 20 mM MG132 for 4h. The lysates were blotted with anti-myc, Flag, and GAPDH antibodies.

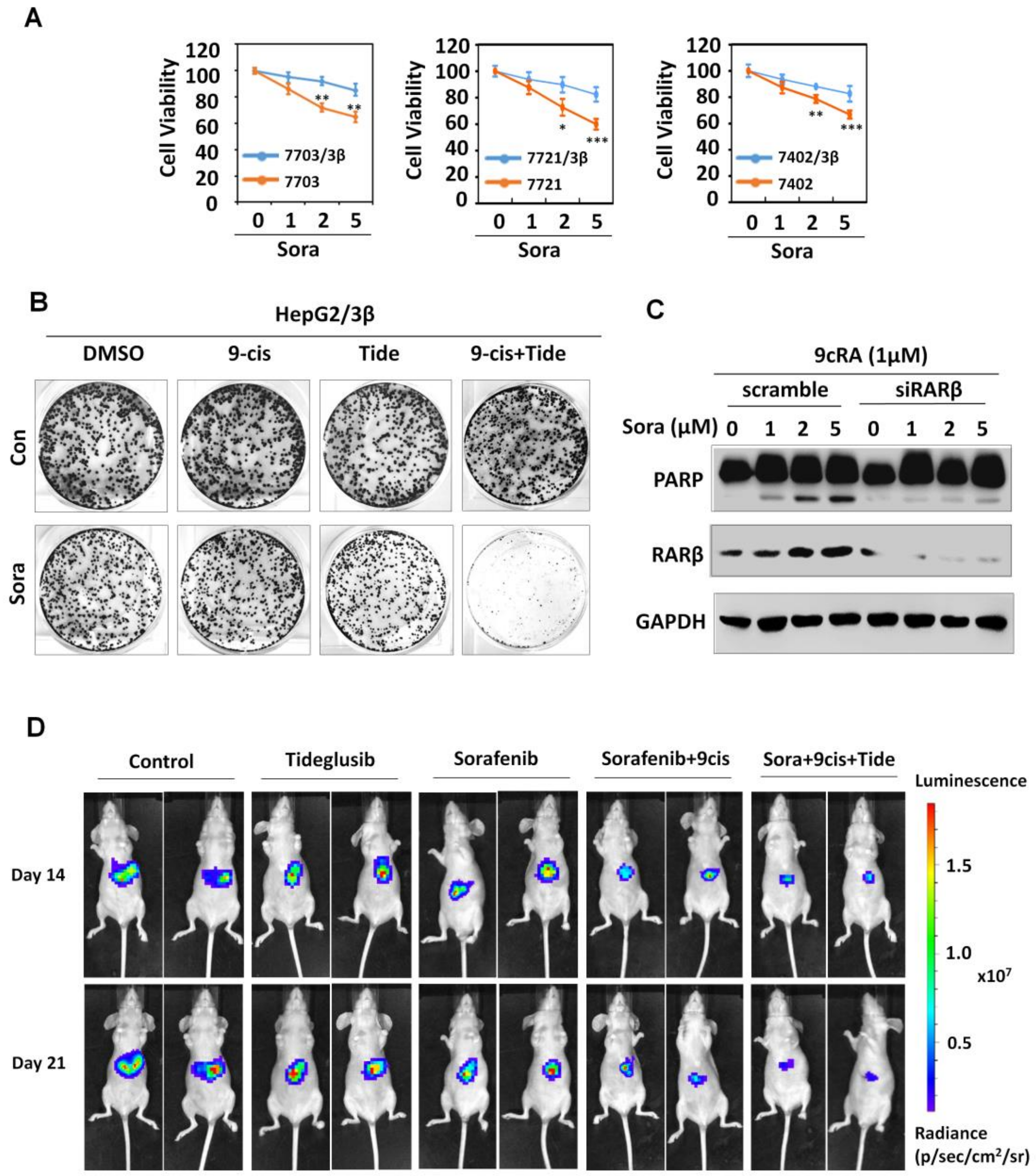

Figure S6. GSK-3β induces sorafenib resistance. (A) MTT assay. Control and GSK-3β stable cell lines were treated with vehicle or with increasing concentrations of sorafenib for 48 h. The cell proliferation was determined with MTT assay with Microplate Reader. **(B)** Colony formation assay. HepG2/3β cells (≤500 cells/well) cultured in 6-well plates were treated with 2 μM sorafenib, 1 μM 9cRA, 2 μM tideglusib, alone or in combination, for 15 days. The cells were fixed in 4% paraformaldehyde and stained with 0.5% crystal violet solution for analysis. (**C**) 9-*cis*-RA enhances sorafenib action through increasing RARβ expression. HepG2 cells were transfected with RARβ siRNA or scramble siRNA for 48 h. The cells were treated with 2 μM 9-*cis*-RA with or without increasing concentrations of sorafenib for 24 h. The lysates were blotted with antibodies against PARP, RARβ and GAPDH. **(D)** Orthotopical tumor. HepG2/3β-luci cells (5×10^5 cells/mice) were injected into hepatic lobular epidermis. After one week, the mice were subjected to different treatments through intraperitoneal injection once every other day for three weeks (n=6 mice per group). The fluorescent images were captured at Day 14 and Day 21. Representative images were shown. Quantitative value upon radiance (p/sec/cm2/sr) indicates the scale and represents the fluorescence intensity.

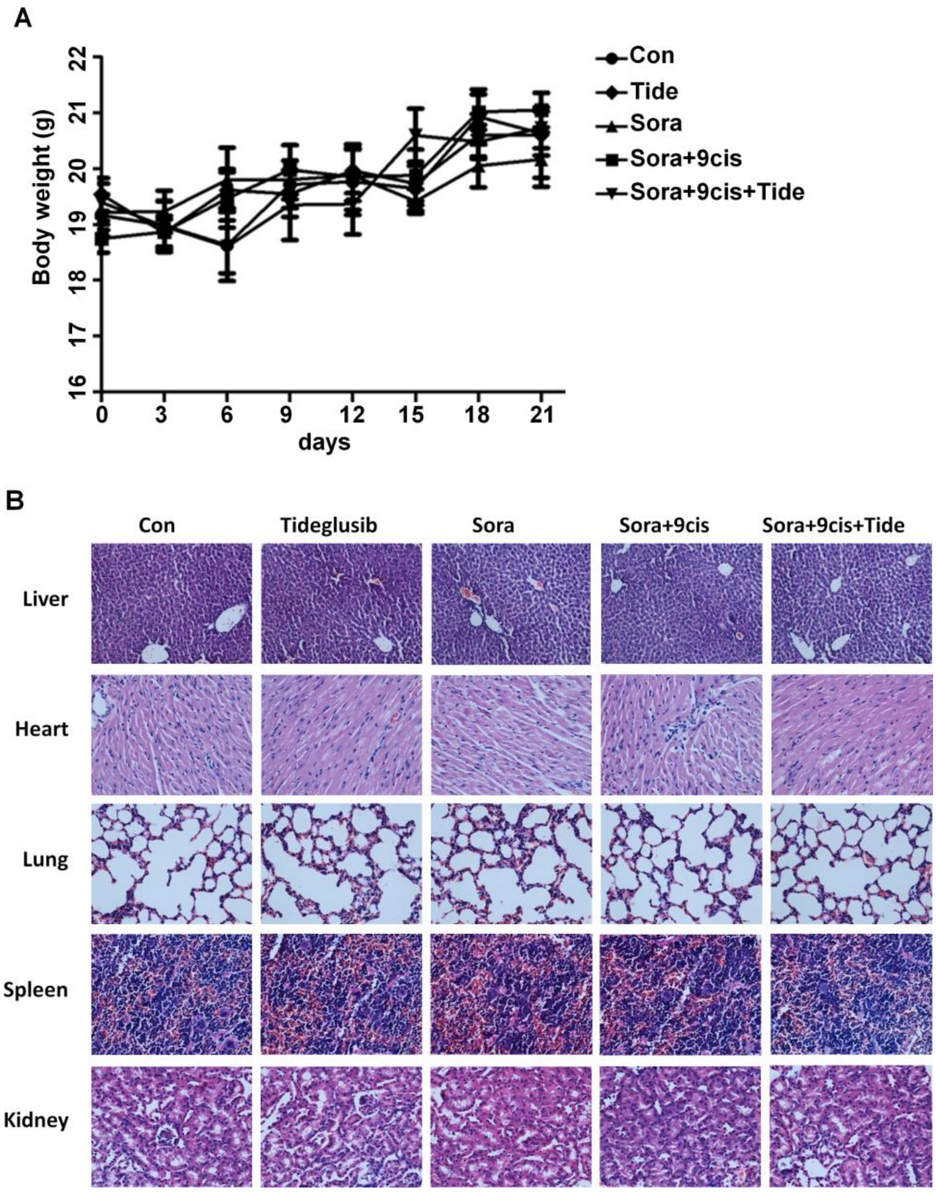

Figure S7. No side effect of combined treatment. (A) Mice weight were measured every 3 days after treatment. The data was analyzed by GraphPad Prism Software. (**B**) The organs were collected and fixed with 4% paraformaldehyde. The paraffin sections were deparaffiized using 100% xylene and 100%, 95%, 85%, 75%, 50% ethanol and stained with hematoxylin and eosin (H&E). The stained sections were analyzed using a Leica microscope (Leica Corporation, Germany).
